# Performance and Metabolic, Inflammatory, and Oxidative Stress-Related Parameters in Early Lactating Dairy Cows with High and Low Hepatic FGF21 Expression

**DOI:** 10.3390/ani13010131

**Published:** 2022-12-29

**Authors:** Denise K. Gessner, Lena M. Sandrock, Erika Most, Christian Koch, Robert Ringseis, Klaus Eder

**Affiliations:** 1 Institute of Animal Nutrition and Nutrition Physiology, Justus Liebig University Giessen, Heinrich-Buff-Ring 26-32, 35392 Giessen, Germany; 2 Educational and Research Centre for Animal Husbandry, Hofgut Neumühle, 67728 Münchweiler an der Alsenz, Germany

**Keywords:** dairy cow, fibroblast growth factor 21, early lactation, ER stress

## Abstract

**Simple Summary:**

Studies with rodent models have shown that fibroblast growth factor 21 (FGF21) is a metabolic regulator induced in the liver in response to different stress conditions, such as energy and nutrient deprivation, inflammation, and metabolic disorders. Recently, it has been found that hepatic *FGF21* expression is strongly upregulated in dairy cows during early lactation. However, the function of FGF21 in cows has not yet been established. Therefore, the aim of the present study was to gain knowledge about the physiological role played by FGF21 in cows during this period. To this end, out of 30 cows, 8 cows with the highest hepatic *FGF21* expression were compared to 8 cows with the lowest hepatic *FGF21* expression. Cows with high and low hepatic *FGF21* expression did not differ in milk yield, feed intake, nor energy balance. Transcriptomics screening, targeted plasma metabolomics, and analyses of antioxidant parameters indicated that high hepatic *FGF21* expression was related to endoplasmic reticulum stress and induction of the antioxidative system in the livers of dairy cows. Therefore, the data of this study suggest that FGF21 plays an important role in the adaptation to cellular stress conditions in early lactation when cows are typically confronted with several stress stimuli.

**Abstract:**

Induction of *FGF21* expression in the liver and a significant increase in plasma FGF21 concentration have been demonstrated in cows during early lactation, but knowledge about the function of FGF21 in dairy cows remains limited. In order to improve the understanding of the physiological role of FGF21 in dairy cows, the present study aimed to investigate differences in metabolic pathways between dairy cows with high and low hepatic expression of *FGF21* at week 1 of lactation (*n* = 8/group) by liver transcriptomics, targeted plasma metabolomics, and analysis of inflammatory and oxidative stress-related parameters. Dry matter intake, energy balance, milk yield, and energy-corrected milk yield at days 8–14 postpartum did not differ between cows with high and low hepatic *FGF21* expression. However, cows with high *FGF21* expression showed an upregulation of genes involved in endoplasmic reticulum stress, inflammation, and nuclear factor E2-related factor 2 (Nrf2)-dependent cytoprotection compared to cows with low *FGF21* expression at week 1 postpartum (*p* < 0.05). Concentrations of important antioxidants (tocopherols, β-carotene, and glutathione) in the liver and plasma, trolox equivalent antioxidant capacity in plasma, concentrations of oxidative stress-related compounds (thiobarbituric acid-reactive substances and protein carbonyls), and levels of most acute phase proteins at week 1 postpartum did not differ between cows with high or low *FGF21* expression. Moreover, among a total of >200 metabolites assayed in the plasma, concentrations of only 7 metabolites were different between cows with high or low *FGF21* expression (*p* < 0.05). Overall, the results showed that cows with high and low *FGF21* hepatic expression had only moderate differences in metabolism, but FGF21 might be important in the adaptation of dairy cows to stress conditions during early lactation.

## 1. Introduction

Fibroblast growth factor 21 (FGF21) is a metabolic regulator produced mainly in the liver [1], which is induced in response to multiple stressors, including energy deprivation, amino acid deprivation, exercise, inflammation, and metabolic disorders, such as obesity. As a consequence of these stress conditions, FGF21 has been demonstrated to preferentially stimulate metabolic pathways that play a central role in energy mobilization in human cells and rodent models, such as lipolysis, gluconeogenesis, and ketogenesis [2,3]. This indicates that the physiological function of FGF21 is to act as a stress hormone that aims to increase the availability of energy substrates in order to cope with the energy-consuming stress response [4].

High-yield dairy cows commonly exhibit a pronounced negative energy balance (NEB) and are frequently exposed to different metabolic (e.g., non-esterified fatty acids (NEFA)) and inflammatory stimuli (e.g., bacterial compounds, inflammatory mediators) during the periparturient phase and early lactation [5,6,7,8]. In line with the role of FGF21 as a stress hormone, a dramatic induction of *FGF21* expression in the liver and marked increase in FGF21 concentration in plasma have been reported in cows at the day of parturition and during early lactation [9,10,11,12,13,14]. This suggests that FGF21 plays a particular physiological role during this phase and is involved in the metabolic adaptation to NEB and stress conditions in dairy cows. Induction of the expression of *FGF21* in the liver of dairy cows at the day of parturition and in the early lactation period is likely caused by increased hepatic uptake of NEFA released from white adipose tissue (WAT) into circulation, because NEFA are potent ligands of peroxisome proliferator-activated receptor α (PPARα), which acts as a transcriptional regulator of *FGF21* [2,15]. Likewise, overfeeding during the dry period, which stimulates lipolysis in WAT and thus increases NEFA levels in plasma during the postpartum phase, causes an increase in FGF21 plasma concentrations in dairy cows [12,16,17]. In addition, evidence indicates that different cellular stress conditions, such as endoplasmic reticulum (ER) stress or inflammation, which frequently occur in the livers of cows during early lactation, cause the induction of hepatic *FGF21* expression [18,19]. In agreement with this, supplementation of polyphenols reduces not only hepatic ER stress but also the expression of *FGF21* in the liver of dairy cows during the first week of lactation [20,21,22].

Several studies with obese and diabetic mouse models have consistently demonstrated that administration of murine or human recombinant FGF21 reduces the fat mass of the body and hepatic fat content by increasing energy expenditure in WAT and brown adipose tissue [23,24,25], increasing the rate of β-oxidation of fatty acids, and reducing the rate of de novo-fatty acid synthesis [26]. In addition, exogenous application of FGF21 to mice administered a high-fat diet improved oral glucose tolerance and insulin sensitivity [27] due to increased secretion of adiponectin from WAT [28]. Moreover, application of FGF21 improved the profile of plasma lipids (decline of low-density lipoprotein cholesterol and triacylglycerols, elevation of high-density lipoprotein cholesterol). While the function of FGF21 in pathologic rodent models has been extensively studied, knowledge about the function of FGF21 in dairy cows is limited, despite existing reports about the factors underlying induction of *FGF21* expression in dairy cows [29].

Thus, in order to improve the understanding of the physiological function of FGF21 in dairy cows, the present study aimed to explore metabolic differences between high-yield dairy cows differing in their hepatic expression level of *FGF21* (high vs. low hepatic *FGF21* expression) in the early postpartum phase using liver transcriptomics, targeted plasma metabolomics, and supplemental analysis of parameters related to inflammation, the antioxidant system, and the occurrence of oxidative stress. 

## 2. Materials and Methods

### 2.1. Animal Experiment

An experiment with 30 Holstein cows with an average parity of 3.06 (±1.27, SD), average body weight of 772 (±75, SD) kg at week 3 antepartum, and milk yield of 10,470 kg in the previous 305-day lactation period was carried out. All procedures described in this study were performed according to the German Animal Welfare Act. The experimental protocol was approved by the official authorities (Provincial Government of Coblenz, Germany, approval number 23 177–07/G15–20–040). The cows were fed a total mixed ration (TMR), as recently described [30]. The TMR offered during the dry period was composed to meet the requirement of crude protein (CP) and net energy of a cow with a body weight of 650 kg and daily dry matter intake of 12 kg. The TMR offered to the cows after parturition was composed to meet the requirement for CP and net energy for a daily milk yield of 34 kg milk, assuming a daily dry matter intake of 22 kg. Feed components were analyzed according to official protocols for feed analysis [31]. Neutral detergent fiber (NDF) and acid detergent fiber (ADF) were analyzed according to the Van Soest method [32]. The net energy lactation (NEL) of the TMR was calculated according to Gesellschaft für Ernährungsphysiologie (GfE) [33]. 

The TMR fed during the dry period contained (per kg DM): 6.5 MJ NEL, 140 g CP, and 383 g NDF; the TMR fed during lactation contained (per kg DM): 6.8 MJ NEL, 166 g CP, and 356 g NDF. The feed intake of the individual cows was recorded using an electronic feeding system (Roughage Intake Control, Insentec B.V., Marknesse, the Netherlands) from day 5 after parturition. 

Within the whole group of 30 cows, 5 cows had to be medically treated due to the occurrence of either mastitis (3 cases), ketosis (1 case), or hypocalcemia (1 case). Among the remaining 25 cows that were not medically treated, 8 cows with the lowest hepatic expression of *FGF21* and 8 cows with the highest *FGF21* expression at week 1 postpartum were selected for further analysis. Thus, *FGF21* expression in the cows was not considered to be influenced by either disease or medical treatment. According to gene expression analysis using GeneChip microarray profiling, the hepatic *FGF21* mRNA level was 4.23-fold higher in the group with high hepatic *FGF21* expression than in the group with low hepatic *FGF21* expression. Based on the results of qPCR analysis, the hepatic *FGF21* expression was 16-fold higher in the group with high hepatic *FGF21* expression than in the group with low hepatic *FGF21* expression. 

### 2.2. Blood and Liver Biopsy Sampling

Blood and liver biopsy samples were taken 2 weeks (12–16 days) before the expected calving date, and at week 1 (days 6–12), week 4 (days 25–32), and week 7 (days 46–52) postpartum. Blood sampling was performed from *V. caudalis mediana* into EDTA-containing vacutainers (S-Monovette 9 mL, Sarstedt, Nümbrecht, Germany) and kept on ice following centrifugation to obtain plasma samples, which were then stored at −80 °C pending analysis. Liver biopsy samples were taken under local anesthesia, as previously described in detail [21]. After removal, the liver biopsy samples were immediately snap-frozen in liquid nitrogen and thereafter stored at −80 °C pending analysis. 

### 2.3. Analysis of Plasma and Liver Samples 

Concentrations of albumin, β-hydroxybutyrate (BHBA), NEFA, total cholesterol, and triacylglycerols (TAG) in plasma were determined using enzymatic reagent kits (Fluitest® ALB, Analyticon, Lichtenfels, Germany; BHBA Assay, Code No. 417–73501, Wako Chemicals GmbH, Neuss, Germany; NEFA Assay, Code No. 436–91995, Wako Chemicals; Fluitest® Chol, Analyticon; Fluitest® TG, Analyticon, Lichtenfels, Germany). Plasma concentrations of haptoglobin (HP), retinol binding protein 4 (RBP4), and serum amyloid A (SAA) were assayed using commercial ELISA kits (EB0011, EB0929b, and EB0015, respectively, Hölzel, Cologne, Germany).

For determination of lipid concentrations in the liver biopsy samples, the lipids were extracted with a mixture of hexane and isopropanol (3:2, *v*/*v*) [34]. Aliquots of the lipid extracts were evaporated. The dried lipids were dissolved in detergent (Triton X-100) [35] and the concentrations of cholesterol and TAG were analyzed using enzymatic reagent kits (Fluitest® CHOL, Fluitest® TG, Analyticon, Lichtenfels, Germany). 

### 2.4. Parameters of Antioxidant Status

The trolox equivalent antioxidative capacity (TEAC) was determined according to the method described by Re et al. [36]. Concentrations of α-tocopherol and β-carotene in the plasma were analyzed by high performance-liquid chromatography according to the method of Balz et al. [37] with slight modifications, as recently described [21]. The plasma concentration of thiobarbituric acid-reactive substances (TBARS) was measured using the method described by Sidwell et al. [38]. The concentration of protein carbonyls in the plasma was measured using the method of Levine et al. [39]. This assay was based on the derivatization of carbonyls with 2,4-dinitrophenylhydrazine (DNPH) to dinitrophenylhydrazone adducts, the extinction of which was proportional to carbonyl content in the sample. The plasma concentration of total glutathione (GSH) was determined by spectrophotometry according to the methods of Tietze [40] and Griffith et al. [41] with slight modifications, as described recently [42]. 

### 2.5. Hepatic GeneChip Microarray Profiling

For this investigation, total RNA was obtained from liver samples taken at week 1 postpartum from 30 cows. Total RNA was extracted as recently described [30]. Processing of total RNA samples was carried out at an Affymetrix service provider, as previously described [22]. The RNA integrity number (RIN) value for all samples was 6.02 ± 0.54 (mean ± SD). In order to identify differentially regulated genes and pathways among animals with the highest and lowest hepatic *FGF21* gene expression, 8 animals with the highest hepatic *FGF21* expression were assigned to the “*FGF21* high” group and 8 animals with the lowest hepatic *FGF21* expression were assigned to the “*FGF21* low” group. Differentially expressed transcripts between the high and low *FGF21* groups were selected based on two filter criteria (fold change (FC) > 1.2 or > −1.2; *p*-value of unpaired Student’s *t*-test < 0.05). Biological meaning from the differentially expressed transcripts was extracted by gene set enrichment analysis (GSEA), as previously described [22], using the DAVID 6.8 bioinformatic resource [43]. The microarray data were deposited in the NCBI Gene Expression Omnibus (GEO accession no: GSE218916).

### 2.6. RNA Extraction and Real-Time Quantitative Polymerase Chain Reaction (qPCR)

The microarray data were validated by qPCR for 25 differentially expressed mRNAs (Supplementary Table S1). Total RNA was subjected to cDNA synthesis and qPCR analysis, as recently described in detail [30]. Primer features are shown in Supplementary Table S2. Calculation of Ct values and normalization of relative gene expression were performed as recently described [22]. The individual GeNorm normalization factor was calculated based on the expression of the three most stable reference genes tested (eukaryotic translation elongation factor 1 alpha 1, H3.3 histone A, and ribosomal protein L12), according to Vandesompele et al. [44]. The presence of a single PCR product was verified by melting curve analysis performed from 50 to 95 °C. The size of the amplified PCR products was checked by agarose gel electrophoresis, as recently described [30]. 

### 2.7. Targeted Metabolite Screening

The concentrations of selected metabolites from different compound classes [21 amino acids, 21 amino acid metabolites, 40 carnitine species, 17 eicosanoids and other oxidation products of polyunsaturated fatty acids (PUFA), 14 lysophosphatidylcholines, 17 oxysterols, 76 phosphatidylcholines, 15 sphingomyelins, and the sum of hexoses] in the plasma of blood samples taken at week 1 postpartum were analyzed using three commercial kits (Absolute/DQ p180 kit, Eicosanoid Assay, and Oxysterol Assay, Biocrates Life Science, Innsbruck, Austria). 

### 2.8. Statistical Analysis

The data were statistically analyzed using the IBM SPSS Statistics v27 software (IBM, Armonk, NY, USA). Normal distribution and homoscedasticity of data were tested by the Shapiro–Wilk test and Levene’s test. Differences between the high and low *FGF21* groups were detected using Student’s *t*-test for normally distributed and homoscedastic data and Welch’s *t*-test for normally distributed and heteroscedastic data. The Kruskal–Wallis test was used for not normally distributed data. Means were considered significantly different for *p* < 0.05. 

## 3. Results

### 3.1. Hepatic FGF21 Expression

**Figure 1:**
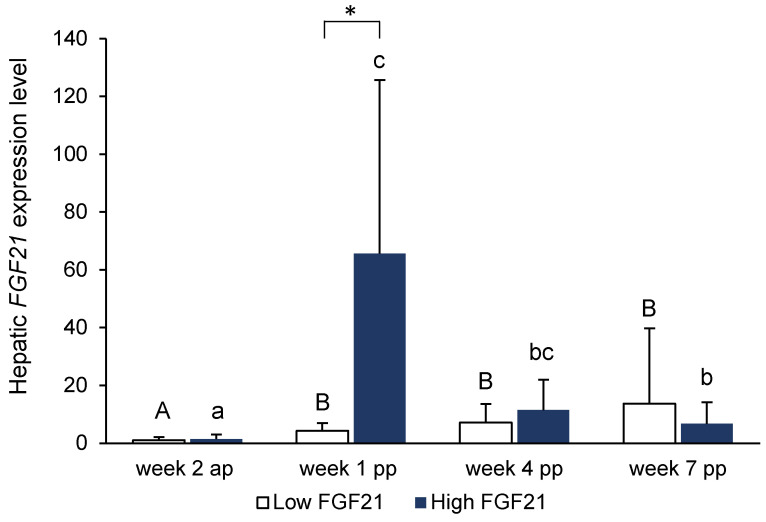
Hepatic *FGF21* expression levels in groups of cows with low versus high hepatic *FGF21* expression at week 2 antepartum, week 1 postpartum, week 4 postpartum, and week 7 postpartum, as analyzed by qPCR (*n* = 8/group). * An asterisk indicates a significant difference between groups. Means without the same uppercase letters (A, B) are significantly different within the low *FGF21* group; means without the same lower case letters (a, b, c) are significantly different within the high *FGF21* group.

Figure 1 shows the relative hepatic gene expression levels of *FGF21* in the cows of the group with low *FGF21* expression and those with high *FGF21* expression at week 2 antepartum and weeks 1, 4, and 7 postpartum, as analyzed by qPCR. In the group with low *FGF21* expression, there was only a moderate increase in *FGF21* expression from week 2 antepartum to week 1 postpartum. In this group, hepatic *FGF21* expression did not decline from week 1 postpartum to weeks 4 and 7 postpartum. In the group with high *FGF21* expression, there was a dramatic increase in *FGF21* expression from week 2 antepartum to week 1 postpartum. Thereafter, *FGF21* expression strongly declined to levels that remained higher than the level at week 2 antepartum. At week 2 antepartum and weeks 4 and 7 postpartum, expression levels of *FGF21* in the liver did not differ between the two groups, but they differed markedly at week 1 postpartum.

### 3.2. Parity and Body Weights of the Cows

The 8 cows with low hepatic *FGF21* expression had an average parity of 2.25 (±0.47, SD), while the 8 cows with high hepatic *FGF21* expression had an average parity of 2.88 (±1.25, SD). The body weights of the two groups did not differ at week 2 antepartum [724 ± 30 (SD) kg for the cows with low hepatic *FGF21* expression, 759 ± 78 (SD) kg for the cows with high hepatic *FGF21* expression], week 1 postpartum [614 ± 35 (SD) kg for the cows with low hepatic *FGF21* expression, 648 ± 54 (SD) kg for the cows with high hepatic *FGF21* expression], week 4 postpartum [589 ± 48 (SD) kg for the cows with low hepatic *FGF21* expression, 642 ± 102 (SD) kg for the cows with high FGF21 hepatic expression], and week 7 postpartum [612 ± 45 (SD) kg for the cows with low hepatic *FGF21* expression, 665 ± 78 (SD) kg for the cows with high hepatic *FGF21* expression]. Body weight changes within the period from week 2 antepartum to week 7 postpartum also did not differ between the two groups.

### 3.3. Feed Intake, Energy Balance and Milk Production

Body weights, dry matter and net energy intake, energy balance, milk yield, and ECM at days 8–14 of lactation did not differ between cows with high and low hepatic *FGF21* expression (Table 1).

### 3.4. Metabolic Parameters in Plasma and Liver

Concentrations of NEFA, BHBA, TAG, and cholesterol in plasma at week 1 postpartum did not differ between cows with high and low hepatic *FGF21* expression (Table 2). Hepatic concentrations of TAG and cholesterol also did not differ between the two groups (Table 2).

### 3.5. Identification of Differentially Expressed Hepatic Genes

**Figure 2:**
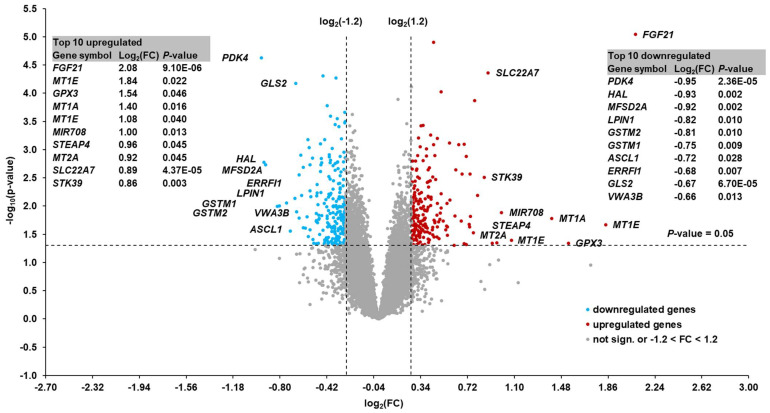
Volcano plot showing hepatic transcripts that were differentially regulated between cows with high and low hepatic *FGF21* expression at week 1 postpartum. The double filtering criteria are indicated by horizontal (*p*-value < 0.05) and vertical [FC: > log_2_(1.2) or < log_2_(−1.2)] dashed lines. Transcripts in the upper left and upper right corners represent the downregulated and upregulated transcripts, respectively. The top 10 up- and downregulated transcripts with log_2_(FC) and *p*-value are shown in the two tables within the figure.

According to the two filter criteria, a total of 410 transcripts were identified as differentially expressed between cows with high and low hepatic *FGF21* expression (Figure 2). Among these genes, 190 were upregulated and 220 were downregulated. Amongst the upregulated genes, only six transcripts were regulated > 2.0-fold. The top 10 upregulated transcripts in the cows with high vs. low hepatic *FGF21* expression were (FC in brackets): *FGF21* (4.23), *MT1E* (3.58), *GPX3* (2.90), *MT1A* (2.64), *MT1E* (2.11), *MIR708* (2.00), *STEAP4* (1.94), *MT2A* (1.89), *SLC22A7* (1.85), and *STK39* (1.81). None of the downregulated transcripts were regulated < −2.0-fold. The top 10 downregulated transcripts in the cows with high vs. low *FGF21* hepatic expression were (FC in brackets): *PDK4* (−1.93), *HAL* (−1.91), *MFSD2A* (−1.89), *LPIN1* (−1.77), *GSTM2* (−1.75), *GSTM1* (−1.68), *ASCL1* (−1.64), *ERRFI1* (−1.60), *GLS2* (−1.59), and *VWA3B* (−1.58). Supplementary Table S3 shows the FC and *p*-values of all transcripts that were differentially expressed between cows with high and low *FGF21* expression.

### 3.6. Technical Validation of Microarray Data for Selected Differentially Expressed Hepatic Genes

The microarray data of 25 transcripts that were expressed differentially were validated by qPCR. Supplementary Table S1 demonstrates that the effect direction (positive or negative FC) could be confirmed by qPCR for all of these transcripts. The effect size (value of FC) differed to some extent for the transcripts validated by microarray and qPCR. Statistical analysis of the qPCR data revealed that 17 of the validated transcripts were significantly regulated (*p* < 0.05). The remaining transcripts were not significantly regulated (*p* ≥ 0.05).

### 3.7. Identification of Biological Processes and Pathways Affected by the Differentially Expressed Hepatic Genes

**Figure 3:**
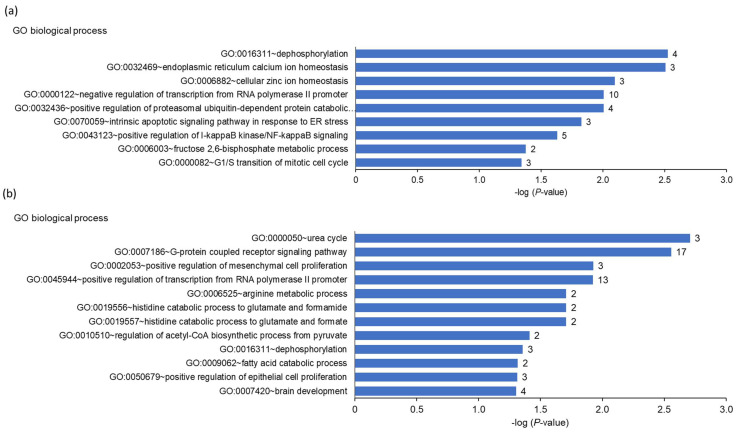

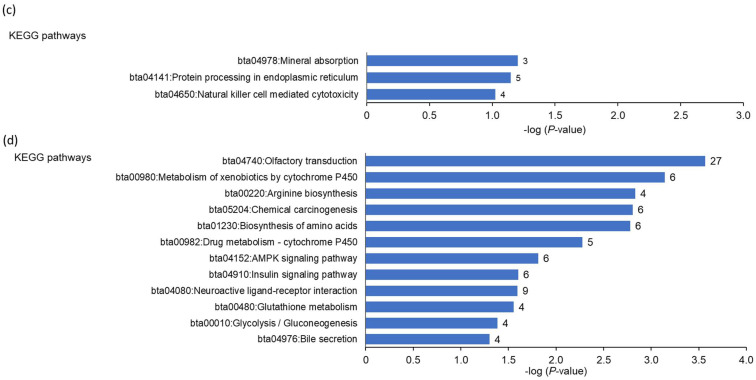
Enriched gene ontology (GO) biological process (**a**,**b**) and KEGG pathways (**c**,**d**) terms assigned to the differentially regulated transcripts between cows with high and low hepatic *FGF21* expression at week 1 postpartum. GO terms and KEGG pathways are sorted by their enrichment *p*-values (EASE score) (top: lowest *p*-value, bottom: highest *p*-value). The number of genes is shown next to the bars.

GSEA of the differentially upregulated transcripts between cows with high and low *FGF21* expression revealed that the most enriched biological process terms included intrinsic apoptotic signaling pathway in response to ER stress, endoplasmic reticulum calcium ion homeostasis, positive regulation of I-kappaB kinase/NF-kappaB signaling, and positive regulation of proteasomal ubiquitin-dependent protein catabolic process, among others (*p* < 0.05, Figure 3a). 

The most enriched biological process terms assigned to the downregulated transcripts between cows with high and low hepatic *FGF21* expression included arginine metabolic process, regulation of acetyl-CoA biosynthetic process from pyruvate, fatty acid catabolic process, and histidine catabolic process to glutamate and formamide, among others (*p* < 0.05, Figure 3b). The enriched KEGG pathways associated with the upregulated transcripts between cows with high and low hepatic *FGF21* expression included mineral absorption and protein processing in the ER, among others (*p* < 0.05, Figure 3c). The most enriched KEGG pathways associated with the downregulated transcripts included olfactory transduction, metabolism of xenobiotics by cytochrome P450, and arginine biosynthesis (Figure 3d).

### 3.8. Hepatic Expression of Genes Involved in Energy Metabolism

To evaluate differences in energy metabolism between cows with high and low hepatic *FGF21* expression, the microarray data were selected for 18 genes involved in mitochondrial and peroxisomal β-oxidation and mitochondrial fatty acid import (*CPT1A, CPT1B, SLC25A20/CACT, ACOX1, ACADS, ACADM, ACADL, ACADVL, ECH1, ECHS1, HADHA, EHHADH, HADH, HADHB, ACAA1, ACAA2, HSD17B4, ACADSB*); 18 genes associated with gluconeogenesis (*PCK1, PCK2, ENO1 to 4, PGAM1, PGAM2, BPGM, PGK1, PGK2, GAPDH, TPI1, ALDOC, ALDOA, ALDOB, FBP1, FBP2);* 8 genes associated with ketogenesis *(ACAT1, ACAT2, HMGCS1, HMGCS2, HMGCL, HMGCLL1, BDH1, BDH2);* and 12 genes associated with the tricarboxylic acid cycle *(ACO1, ACO2, CS, DLD, DLST, FH, IDH1, IDH2, IDH3A, IDH3B, IDH3G, MDH1, MDH2, OGDH, OGDHL, SDHA, SDHB, SDHC, SDHD*; *SUCLG1, SUCLG2) (*Table 3). While the vast majority of these genes were not significantly regulated, 4 (*EHHADH, HADH, ACAA2, HSD17B4*) out of 18 genes involved in β-oxidation and mitochondrial fatty acid import, 4 (*ENO2, ENO4, ALDOB, FBP1*) out of 18 genes involved in gluconeogenesis, 1 (*HMGCS1*) out of 8 genes involved in ketogenesis, and 5 (*CS, IDH3B, OGDHL, SUCLG2, MDH2*) out of 21 genes involved in tricarboxylic acid were significantly regulated (*p* < 0.05).

### 3.9. Hepatic Expression of Genes Involved in Inflammation and Stress Response

To assess differences in inflammation and stress response between cows with high and low hepatic *FGF21* expression, the microarray data were selected for 15 genes involved in inflammation (*CCL2, CP, CRP, CXCL8, FYN, HP, IFNG, IL1B, IL6, LITAF, PTGS2, SAA2, SAA3, SAA4, TNF*); 19 genes associated with ER stress (*ATF4, ATF6, BAK1, BAX, CASP3, CASP8, CASP9, CHAC1, DDIT3, DNAJC3, EDEM1, FGF21, HERPUD1, HSP90B1, HSPA5, PDIA4, PPP1R15A, TRIB3, XBP*1); and 20 genes associated with Nrf2-dependent cytoprotection (*CAT, GCLC, GCLM, GPX1, GPX3, GSTA2, HMOX1, MT1A, MT2A, MT1E, MT3, MT4, NFE2L2, NQO1, SOD1, SRXN1, TXN, TXNRD1, UGT1A1, UGT1A6*) (Table 4). Amongst these genes, 3 genes (*FYN, LITAF, SAA4*) involved in inflammation and 7 genes (*ATF4, CHAC1, DDIT3, FGF21, HERPUD1, HSPA5, TRIB3*) involved in ER stress were upregulated in cows with high hepatic *FGF21* expression compared to cows with low hepatic *FGF21* expression (*p* < 0.05). Amongst the Nrf2-dependent genes, 4 genes (*GPX3, MT1A, MT1E, MT2A*) were upregulated and 3 genes (*CAT, MT4, GSTA2*) were downregulated in cows with high hepatic *FGF21* expression compared to cows with low hepatic *FGF21* expression (*p* < 0.05).

### 3.10. Identification of Differentially Regulated Plasma Metabolites

**Figure 4:**
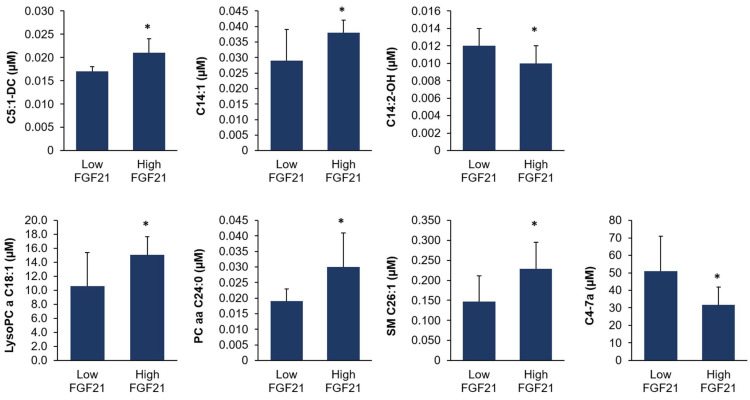
Concentrations of plasma metabolites that were identified by targeted metabolomics analysis to be different between cows with high and low hepatic *FGF21* expression at week 1 postpartum. Bars represent means ± SD, *n* = 8 for each group. Abbreviations: a, acyl residue; aa, acyl-acyl residue; C4–7a, 7-alpha-hydroxy-cholestenone; C5:1-DC; glutaconylcarnitine; C14:1, tetradecenoylcarnitine; C14:2-OH, hydroxytetradecadienylcarnitine; Lyso PC, lysophosphatidylcholine; PC, phosphatidylcholine; SM, sphingomyelin. * An asterisk indicates a significant difference between the two groups (*p* < 0.05).

Targeted metabolomics analysis was carried out to detect plasma metabolites differing between cows with high and low hepatic *FGF21* expression at week 1 postpartum. The quantification of >200 metabolites of various classes of compounds revealed only seven plasma metabolites with differing concentrations between the two groups (Figure 4, *p* < 0.05). These metabolites were members of the classes acylcarnitines (C5:1-DC, C14:1, C14:2-OH), glycerophospholipids (lyso-PC a C18:1, PC aa C24:0), sphingolipids (SM_C26:1), and sterols (C4_7a). The concentrations of five metabolites (C5:1-DC, C14:1, lysoPC a C18:1, PC aa C24:0, SM_C26:1) were higher in the cows with high *FGF21* expression and those of the remaining two metabolites (C14:2-OH, C4_7a) were lower in the cows with high *FGF21* expression than in the cows with low *FGF21* expression (*p* < 0.05).

### 3.11. Parameters of Antioxidant Status in Plasma and Liver

The concentrations of important antioxidants (tocopherols, β-carotene, GSH) in the liver and/or plasma did not differ between cows with high and low hepatic *FGF21* expression (Table 5). In line with this, plasma concentrations of TEAC and some oxidative stress-related parameters (TBARS, protein carbonyles) did not differ between cows with high and low hepatic *FGF21* expression (Table 5).

### 3.12. Concentrations of Acute Phase Proteins and Oxylipids in Plasma

The plasma concentrations of two positive (HP, SAA) and two negative acute phase proteins (albumin, RBP4) were determined. While the concentration of SAA was higher in cows with high hepatic *FGF21* expression than in cows with low hepatic *FGF21* expression (*p* < 0.05), the concentrations of the other acute phase proteins did not differ between the two groups of cows (Table 6). Plasma concentrations of oxylipids derived from enzymatic and non-enzymatic oxidation of linoleic acid (9-HODE, 13-HODE) and arachidonic acid (12-HETE, 15-HETE, LTB4, PGF2α) also did not differ between cows with high and low hepatic *FGF21* expression (Table 6).

## 4. Discussion

In order to gain a deeper understanding of the role played by FGF21 in dairy cows during early lactation, this study compared performance and metabolic, inflammatory, and oxidative stress-related parameters in two groups of high-yield dairy cows that markedly differed in their hepatic expression levels of *FGF21* at week 1 postpartum. This time-point was chosen for two main reasons. First, the finding that the expression of *FGF21* is dramatically upregulated and plasma FGF21 concentrations are strongly increased during the first few days after parturition and strongly decline thereafter suggests that FGF21 exerts its main biological functions in dairy cows during the first week postpartum [9,10,11,12,13,45]. Second, based on the suggestion that FGF21 acts as a stress hormone released from the liver to cope with stress conditions [4], we expected that the biological effects of FGF21 would be most prominent during the phase in which cellular stress is most pronounced. Previous studies have shown that stress conditions, such as ER stress or inflammation, are most enhanced during the first week after parturition and strongly decline thereafter [46,47]. At week 5 postpartum, stress conditions such as ER stress and inflammation are already nearly absent [46,47,48]. 

Analysis of *FGF21* expression in the liver in a cohort of 30 cows in our study demonstrated that the increase in *FGF21* expression at week 1 postpartum showed great individual variation. While some cows showed a dramatic increase compared to the antepartum level, hepatic *FGF21* expression in others remained nearly unchanged. Our study, however, confirmed the findings of other studies in which cows with strongly increased expression levels of *FGF21* in early lactation showed strongly declining expression levels towards later lactation [10,11]. In order to identify factors that could be involved in the expression of *FGF21* and to figure out a possible role of FGF21 in metabolic regulation, we selected the 8 cows with the highest hepatic *FGF21* expression and the 8 cows with the lowest expression at week 1 postpartum out of a cohort of 30 cows, and assigned them to two groups. Performance parameters, such as dry matter intake, net energy intake, milk yield, and ECM, body weights, and energy balance did not differ between these two groups at days 8–14 postpartum; thus, it can be excluded that the performance level or energy balance of the cows caused the differential expression of hepatic *FGF21* in these two groups. Although the strong induction of *FGF21* expression in the liver of dairy cows in early lactation is considered to be caused by the pronounced hepatic uptake of NEFA released from WAT during this phase [10,11,13], the stronger induction of *FGF21* expression in the cows with high hepatic *FGF21* expression was probably not due to greater intrahepatic availability of NEFA because plasma concentrations of NEFA did not differ between the groups. Moreover, other metabolic parameters characterizing energy balance and hepatic lipid metabolism, such as plasma concentrations of BHBA, cholesterol, and TAG, and hepatic concentrations of cholesterol and TAG, did not differ between the two groups. This clearly indicated that the marked difference in hepatic *FGF21* expression between the two groups of cows was not caused by differences in energy mobilization and hepatic lipid metabolism. The finding that body weight loss during the early lactation period also did not differ between the two groups of cows supported the indication that the difference in hepatic *FGF21* expression was not due to a different rate of body fat mobilization.

In order to identify metabolic pathways influenced by hepatic *FGF21* expression at week 1 postpartum, we performed transcriptomics analysis using microarray technology. The RNA integrity measurement indicates the presence of some kind of RNA degradation that can limit sensitivity (the rate of detection of true positives among all positives) and specificity (the rate of detection of true negatives among all negatives) of microarray performance [49]. Nevertheless, the mean RIN value >6 suggested that the RNA integrity was sufficient to carry out microarray hybridizations. In addition, the microarray analysis was primarily used as a screening technique to identify changes in sets of pathway-specific genes, rather than as a tool to accurately quantify the expression of differentially regulated genes. To obtain deeper insight into the main metabolic pathways of energy metabolism that might differ between the two groups, the microarray expression levels of genes associated with β-oxidation and mitochondrial fatty acid import, gluconeogenesis, ketogenesis, and tricarboxylic acid were evaluated. Due to the low number of selected cows with low and high hepatic *FGF21* expression (*n* = 8), this approach was preferred rather than carrying out principal component analysis or partial least-squares discrimination analysis, both of which require larger sample sizes to detect differences in discriminant genes. Our analysis showed that very few of these genes were differentially regulated between cows with high and low hepatic *FGF21* expression and the regulation of these genes was moderate [FC between −1.32 (*FBP1*) and +1.34 (*CS*)]. In order to determine whether *FGF21* expression in the liver was related to alterations on a metabolic level, we performed targeted plasma metabolomics analysis. We observed that the concentrations of only seven out of >200 metabolites were significantly different between the two groups of cows. These seven metabolites belonged to four different metabolite classes (acylcarnitines, glycerophospholipids, sphingolipids, and sterols). Acylcarnitines play a role in the β-oxidation of fatty acids within the mitochondrion. However, acylcarnitines represented only minor species with differing plasma concentrations between the two groups of cows and the concentrations of total carnitine and acetylcarnitine in plasma did not differ between the two group of cows. Therefore, there was no overall indication that *FGF21* expression affected the β-oxidation of fatty acids by influencing carnitine metabolism. In a similar manner, the three individual phospholipid species and sterol derivative with differing plasma concentrations between the two groups of cows were minor components, suggesting that *FGF21* expression was not linked to substantial effects on phospholipid and sterol metabolism. The observation that the small number of regulated plasma metabolites belonged to four different metabolite classes was a further indication that the hepatic expression level of *FGF21* was not associated with the consistent regulation of a specific metabolic pathway. This showed that cows with high and low hepatic *FGF21* expression did not differ with regard to hepatic energy metabolism, indicating that other factors must be responsible for the marked difference in hepatic *FGF21* expression. Likewise, the application of exogenous FGF21 had no effect on fatty acid metabolism in dairy cows [50]. In addition, FGF21 administration in dairy cows did not affect plasma concentrations of insulin and glucose and insulin concentrations in a glucose tolerance test [51], indicating that glucose metabolism was also not affected by FGF21 in dairy cows. An important reason for the lack of effect of exogenous FGF21 and lack of differences in the main metabolic pathways of energy metabolism between cows with high and low hepatic *FGF21* expression might be that the liver, unlike WAT, is not the primary tissue targeted by the action of FGF21. This is reflected by the fact that the physiologic FGF21 receptor FGFR1c, which is strongly expressed in WAT, is nearly absent in cattle liver whereas β-klotho, the co-activator of FGF21, is expressed in cattle liver at a markedly lower level than in WAT [10].

Previous studies have shown that dairy cows are subject to ER stress during early lactation, a phenomenon that could be involved in the development of fatty liver and metabolic diseases during this period [47,52,53,54]. With respect to this finding, an interesting observation from the liver transcriptomics of the present study is that the upregulated transcripts in the cows with high hepatic *FGF21* expression were involved particularly in the ER stress-induced unfolded protein response (UPR). This was evident from the gene set enrichment analysis, which demonstrated that the intrinsic apoptotic signaling pathway in response to ER stress, response to nutrient levels, response to extracellular stimulus, response to starvation, programmed cell death, and protein processing in the ER were amongst the most enriched biological process terms and KEGG pathways within the genes upregulated in the cows with high hepatic *FGF21* expression. In line with this, the upregulated transcripts in cows with high hepatic *FGF21* expression included several typical ER stress-target genes, such as *ATF4, HERPUD1, HSPA5, DDIT3, WFS1, DNAJB11, CHAC1,* and *TRIB3*. ER stress is well-known to be activated as a consequence of different stress conditions, such as nutrient deprivation, inflammation, and oxidative stress, which cause imbalances in ER quality control pathways leading to the accumulation of unfolded or misfolded proteins in the ER [55]. In order to combat ER stress, mammalian cells are equipped with a defense system, called UPR, which enables the cell to re-establish ER quality control and decrease the accumulation of unfolded or misfolded proteins in the ER [56]. Apart from protein kinase RNA-like ER kinase (PERK)-dependent transient attenuation of new protein synthesis and stimulation of inositol-requiring protein 1a (IRE1)-dependent mRNA degradation, another important UPR mechanism is the targeting of unfolded or misfolded proteins towards the ER-associated degradation pathway, where they are transferred to the cytosol and degraded by the ubiquitin proteasome system (UPS) [57]—the most important pathway of intracellular protein degradation. In agreement with this UPR mechanism, the gene set enrichment analysis also revealed that regulation of protein catabolic process, positive regulation of proteasomal ubiquitin-dependent protein catabolic process, regulation of protein metabolic process, and positive regulation of proteasomal protein catabolic process were overrepresented terms amongst the genes upregulated in the cows with high hepatic *FGF21* expression. Remarkably, a recent study convincingly demonstrated that FGF21 plays an important role in muscle atrophy during fasting, reporting that the muscle mass of wild-type mice was significantly reduced in response to fasting, whereas muscle-specific FGF21 knockout mice were protected against muscle loss and weakness during fasting [58]. In addition, the authors showed that in vivo FGF21 overexpression in skeletal muscle induced muscle atrophy, thus supporting a role for FGF21 in skeletal muscle proteolysis. Despite that the authors of this study did not study a possible involvement of ER stress, their results indicated that *FGF21* expression was associated with increased protein catabolism.

If ER stress is overwhelming and ER homeostasis cannot be restored, the UPR can also activate signaling pathways that initiate programmed cell death. Convincing evidence has been gained from cell culture studies and studies with laboratory animals that ER stress-induced UPR leads to an upregulation of hepatic *FGF21* expression through the activation of ER stress-transducers PERK and IRE1 [18,19]. Based on these findings, it is likely that ER stress was responsible for the strong induction of *FGF21* expression in the cows with high hepatic *FGF21* expression. Previous observations with dairy cows, in which polyphenol-enriched feeding rations decreased not only the hepatic expression of UPR-associated genes but also the expression of FGF21 [20,21,22], are supportive of such a relationship between hepatic ER stress and FGF21 production. 

Activation of nuclear factor E2-related factor 2 (Nrf2) in the liver of dairy cows during early lactation was shown in a previous study [46]. Nrf2 is a transcription factor that regulates the expression of a broad range of antioxidant and cytoprotective genes [59]. Hepatic transcript profiling in the present study revealed the upregulation of several target genes of Nrf2, including *GPX3, MT1A, MT2A,* and *MT1E*, in cows with high hepatic *FGF21* expression. Because activation of Nrf2 signaling is a known downstream event of ER stress, which aims to counteract oxidative stress and inflammatory conditions which are frequently associated with ER stress [60,61], induction of Nrf2-dependent genes is likely a consequence of the induction of ER stress-related genes and a further indicator of the occurrence of ER stress in the cows with high hepatic *FGF21* expression. It has been well established that ER stress is linked to the induction of pro-inflammatory conditions [62]. Therefore, we also considered genes associated with inflammation in the liver and concentrations of acute phase proteins in plasma. We observed that there was an up-regulation of two genes encoding serum amyloid A (*SAA2, SAA4*) in the liver and an increased concentration of SAA in plasma of the cows with high *FGF21* expression in the liver compared to cows with low *FGF21* expression in the liver. SAA belongs to the group of acute phase proteins produced in the liver and released into the blood in the course of inflammation [63]. Therefore, increased production of SAA in the liver of the group of cows with high hepatic *FGF21* expression indicates that these cows could have suffered pro-inflammatory conditions in the liver. However, the expression of several other genes encoding proteins involved in inflammation (such as *TNF*) and acute phase reaction (*CP*, *HP*) in the liver did not differ between the two groups of cows, indicating that the increased expression of *FGF21* was not related to the development of a pro-inflammatory condition. Moreover, plasma concentrations of oxylipids, including 9-HODE, 13-HODE, 12-HETE, 15-HETE, LTB4, and PGF2α, also did not differ between the two groups of cows. Oxylipids are a class of lipid mediators produced by enzymatic and non-enzymatic oxidation of PUFA, dominantly linoleic acid (LA) and arachidonic acid (AA). LA-derived 9-HODE and 13-HODE, but also AA-derived HETEs and eicosanoids, promote inflammatory responses by acting as chemoattractants for circulating immune cells [64]. Thus, the present data suggest that the cows with high hepatic *FGF21* expression did not exhibit a systemic inflammatory process. In addition, the cows with high and low hepatic *FGF21* expression did not differ with regard to the plasma concentrations of antioxidants, such as tocopherols, β-carotene, and GSH, and indicators of antioxidant status, such as TEAC, TBARS, and protein carbonyls. This indicated that the cows with high hepatic *FGF21* expression also did not experience systemic oxidative stress, which is frequently associated with inflammation. Albeit speculative, it is possible that the induction of Nrf2-dependent genes observed in the cows with high hepatic *FGF21* expression prevented the development of systemic oxidative stress, because activation of Nrf2 signaling activated the expression of genes encoding cytoprotective enzymes, including antioxidant enzymes, thereby providing protection against ROS generated during the inflammatory process. This assumption is supported by recent evidence that FGF21 inhibits oxidative stress by stimulating Nrf2-dependent induction of cytoprotective and antioxidative genes [65,66,67]. This would suggest that FGF21 plays an important role in the adaptation to cellular stress conditions in dairy cows during early lactation—a phase during which high-yield dairy cows are confronted with several different stress stimuli [5,6,7,8]. Indeed, our assumption of FGF21 as a stress-modulating factor in early-lactation dairy cows is supported by the results from a mice study that suggested that *FGF21* induction was a potential strategy to protect against the toxicity resulting from experimentally induced sepsis [68]. While the upregulated genes in the cows with high *FGF21* expression were found to be involved particularly in ER stress-induced UPR, the downregulated genes in the cows with high hepatic *FGF21* expression were identified to play a role in the cellular amino acid catabolic process, alpha-amino acid metabolic process, glutamine family amino acid metabolic process, arginine biosynthesis, and urea cycle. This indicated that hepatic amino acid catabolism was attenuated in the cows with high compared to low hepatic *FGF21* expression. Although speculative, this might be interpreted as a mechanism to provide amino acids for specific organ functions, such as acute phase protein synthesis, which are activated as a consequence of ER stress, as seen in the cows with high hepatic *FGF21* expression. Overall, our data indicate that induction of hepatic *FGF21* in cows after parturition (1 week postpartum) might be, at least in part, caused by cellular stress such as ER stress. In turn, FGF21 might induce hepatic signaling pathways, such as Nrf2, which helps the body to cope with stress conditions and protect the liver against damage induced by the inflammation process and increased generation of reactive oxygen species commonly observed in dairy cows after parturition [8,69]. 

## 5. Conclusions

Analysis of *FGF21* expression levels in the livers of a cohort of 30 cows demonstrated that the increase in *FGF21* expression in early lactation (week 1 postpartum) showed a great of individual variation. We observed that the performance data of cows with high and low hepatic *FGF21* expression did not differ at days 8 –14 postpartum, indicating that animal performance level and energy balance are not major determinants of hepatic *FGF21* expression. However, we observed that the *FGF21* expression level was associated with the expression of genes related to ER stress and Nrf2 signaling. These findings suggest that hepatic *FGF21* expression might be induced by ER stress. Upregulation of Nrf2-dependent genes in cows with high hepatic *FGF21* expression might be a compensatory means to combat cellular stress. The finding that the expression of genes involved in metabolic pathways of energy generation (β-oxidation and mitochondrial fatty acid import, gluconeogenesis, ketogenesis, and the tricarboxylic acid cycle) in the liver was not largely different between the two groups of cows indicated that hepatic *FGF21* expression had less overall impact on energy metabolism. This suggestion was supported by the finding that the concentrations of only 7 (out of >200) plasma metabolites from different compound classes differed between cows with high or low hepatic *FGF21* expression. Overall, in line with observations in other animal species, FGF21 seems to have an important function in the adaptation to cellular stress conditions in cows during early lactation, a time period in which cows are commonly confronted with several different stress stimuli.

## Figures and Tables

**Table 1:** Body weights, feed intake, energy balance, and milk performance of cows with high and low hepatic *FGF21* expression at days 8–14 postpartum.

	Low *FGF21*	High *FGF21*	*p*-Value
Body weight, kg	615 ± 27	648 ± 63	0.187
Dry matter intake, kg/d	14.2 ± 4.5	14.9 ± 1.7	0.779
Net energy intake, MJ/d	99.7 ± 31.3	104.4 ± 13.5	0.790
Energy balance, MJ NEL/d	−62.1 ± 18.1	−75.6 ± 31.4	0.484
Milk yield, kg/d	37.5 ± 3.6	36.3 ± 5.3	0.613
ECM, kg/d	40.2 ± 5.7	42.7 ± 8.6	0.549

ECM = energy-corrected milk, adjusted to 40 g fat/kg and 34 g protein/kg; data represent means ± SD, *n*  =  8 for each group.

**Table 2:** Plasma and liver concentrations of metabolic parameters in cows with high and low hepatic *FGF21* expression in week 1 postpartum.

	Low *FGF21*	High *FGF21*	*p*-Value
Plasma			
NEFA, µmol/L	577 ± 245	507 ± 305	0.623
BHBA, mmol/L	1.06 ± 0.486	1.08 ± 0.53	1.000
TAG, µmol/L	111 ± 21.9	98.6 ± 16.3	0.238
Cholesterol, µmol/L	2.58 ± 1.05	2.17 ± 0.48	0.462
Liver			
TAG, µmol/g	27.4 ± 18.3	35.7 ± 17.2	0.363
Cholesterol, µmol/g	3.25 ± 0.70	2.98 ± 0.49	0.406

NEFA = Non-esterified fatty acids; BHBA = β-hydroxybutyrate; TAG = triacylglycerol; data represent means ± SD, *n* = 8 for each group.

**Table 3:** Expression [fold change (FC)] of hepatic genes involved in β-oxidation and mitochondrial fatty acid uptake, gluconeogenesis, ketogenesis, and the tricarboxylic acid cycle in cows with high vs. low hepatic *FGF21* expression at week 1 postpartum.

Gene Symbol	Gene Description	FC	*p*-Value
*β-oxidation and mitochondrial fatty acid uptake*			
*ACAA1*	Acetyl-CoA acyltransferase 1	0.95	0.447
*ACAA2*	Acetyl-CoA acyltransferase 2	−1.13	0.006
*ACADL*	Acyl-CoA dehydrogenase long chain	0.97	0.640
*ACADM*	Acyl-CoA dehydrogenase medium chain	0.97	0.438
*ACADS*	Acyl-CoA dehydrogenase short chain	0.94	0.341
*ACADSB*	Acyl-CoA dehydrogenase short/branched chain	1.02	0.776
*ACAA1*	Acetyl-CoA acyltransferase 1	0.95	0.447
*ACADVL*	Acyl-CoA dehydrogenase very long chain	0.99	0.910
*ACOX1*	Acyl-CoA oxidase 1	0.97	0.623
*CPT1A*	Carnitine palmitoyltransferase 1A	0.88	0.103
*CPT1B*	Carnitine palmitoyltransferase 1B	1.09	0.629
*ECH1*	Enoyl-CoA hydratase 1	1.07	0.310
*ECHS1*	Enoyl-CoA hydratase, short chain 1	0.95	0.233
*EHHADH*	Enoyl-CoA hydratase and 3-hydroxyacyl CoA dehydrogenase	−1.10	0.029
*HADH*	Hydroxyacyl-CoA dehydrogenase	−1.19	0.007
*HADHA*	Hydroxyacyl-CoA dehydrogenase trifunctional multienzyme complex subunit alpha	0.97	0.751
*HADHB*	Hydroxyacyl-CoA dehydrogenase trifunctional multienzyme complex subunit beta	0.97	0.712
*HSD17B4*	Hydroxysteroid 17-beta dehydrogenase 4	−1.11	0.007
*SLC25A20*	Solute carrier family 25 member 20 (Previous name: carnitine/acylcarnitine translocase)	1.06	0.518
*Gluconeogenesis*			
*ALDOA*	Aldolase, fructose-bisphosphate A	0.94	0.172
*ALDOB*	Aldolase, fructose-bisphosphate B	−1.11	0.024
*ALDOC*	Aldolase, fructose-bisphosphate C	0.88	0.163
*BPGM*	Bisphosphoglycerate mutase	0.97	0.370
*ENO1*	Enolase 1	0.94	0.326
*ENO2*	Enolase 2	−1.22	0.022
*ENO3*	Enolase 3	1.04	0.657
*ENO4*	Enolase 4	1.16	0.018
*FBP1*	Fructose-bisphosphatase 1	−1.32	0.001
*FBP2*	Fructose-bisphosphatase 2	1.30	0.402
*GAPDH*	Glyceraldehyde-3-phosphate dehydrogenase	0.98	0.678
*PCK1*	Phosphoenolpyruvate carboxykinase 1	1.00	0.986
*PCK2*	Phosphoenolpyruvate carboxykinase 2	1.01	0.944
*PGAM1*	Phosphoglycerate mutase 1	0.90	0.168
*PGAM2*	Phosphoglycerate mutase 1	0.95	0.461
*PGK1*	Phosphoglycerate kinase 1	1.01	0.948
*PGK2*	Phosphoglycerate kinase 2	1.07	0.189
*TPI1*	Triosephosphate isomerase 1	0.93	0.258
*Ketogenesis*			
*ACAT1*	Acetyl-CoA acetyltransferase 1	0.90	0.058
*ACAT2*	Acetyl-CoA acetyltransferase 2	1.00	0.983
*BDH1*	3-Hydroxybutyrate dehydrogenase 1	0.91	0.369
*BDH2*	3-Hydroxybutyrate dehydrogenase 2	1.13	0.362
*HMGCL*	3-Hydroxy-3-methylglutaryl-CoA lyase	0.98	0.632
*HMGCLL1*	3-Hydroxymethyl-3-methylglutaryl-CoA lyase like 1	0.99	0.902
*HMGCS1*	3-Hydroxy-3-methylglutaryl-CoA synthase 1	1.21	0.248
*HMGCS2*	3-Hydroxy-3-methylglutaryl-CoA synthase 2	0.93	0.447
*Tricarboxylic acid cycle*			
*ACO1*	Aconitase 1	1.02	0.558
*ACO2*	Aconitase 2	0.94	0.266
*CS*	Citrate synthase	1.34	0.004
*DLD*	Dihydrolipoamide dehydrogenase	0.95	0.282
*DLST*	Dihydrolipoamide S-succinyltransferase	0.92	0.185
*FH*	Fumarate hydratase	0.97	0.695
*IDH1*	Isocitrate dehydrogenase (NADP(+)) 1	0.99	0.835
*IDH2*	Isocitrate dehydrogenase (NADP(+)) 2	1.17	0.111
*IDH3A*	isocitrate dehydrogenase (NAD(+)) 3 catalytic subunit alpha	1.05	0.314
*IDH3B*	Isocitrate dehydrogenase (NAD(+)) 3 non-catalytic subunit beta	−1.11	0.027
*IDH3G*	Isocitrate dehydrogenase (NAD(+)) 3 non-catalytic subunit gamma	0.95	0.134
*MDH1*	Malate dehydrogenase 1	0.94	0.465
*MDH2*	Malate dehydrogenase 2	−1.09	0.017
*OGDH*	Oxoglutarate dehydrogenase	0.82	0.090
*OGDHL*	Oxoglutarate dehydrogenase L	−1.12	0.038
*SDHA*	Succinate dehydrogenase complex flavoprotein subunit A	0.93	0.251
*SDHB*	Succinate dehydrogenase complex iron sulfur subunit B	−1.10	0.036
*SDHC*	Succinate dehydrogenase complex subunit C	0.88	0.172
*SDHD*	Succinate dehydrogenase complex subunit D	0.90	0.076
*SUCLG1*	Succinate-CoA ligase GDP/ADP-forming subunit alpha	0.89	0.109
*SUCLG2*	Succinate-CoA ligase GDP-forming subunit beta	−1.12	0.005

*n* = 8 for each group.

**Table 4:** Expression [fold change (FC)] of hepatic genes involved in inflammation, endoplasmic reticulum (ER) stress/unfolded protein response (UPR), and nuclear factor erythroid 2-related factor 2 (Nrf2)-dependent cytoprotection in cows with high vs. low hepatic *FGF21* expression at week 1 postpartum.

Gene Symbol	Gene Description	FC	*p*-Value
*Inflammation*			
*CCL2*	C-C motif chemokine ligand 2	0.87	0.206
*CP*	Ceruloplasmin	0.93	0.180
*CRP*	C-reactive protein	1.04	0.516
*CXCL8*	C-X-C motif chemokine ligand 8	0.99	0.950
*FYN*	FYN proto-oncogene, Src family tyrosine kinase	1.24	0.002
*HP*	Haptoglobin	3.29	0.110
*IFNG*	Interferon gamma	1.09	0.326
*IL1B*	Interleukin 1 beta	1.27	0.055
*IL6*	Interleukin 6	1.12	0.486
*LITAF*	Lipopolysaccharide induced TNF factor	1.23	0.024
*PTGS2*	Prostaglandin-endoperoxide synthase 2	1.09	0.425
*SAA2*	Serum amyloid A2	1.33	0.069
*SAA3*	Serum amyloid A3, pseudogene	1.78	0.215
*SAA4*	Serum amyloid A4, constitutive	1.64	0.048
*TNF*	Tumor necrosis factor	1.01	0.927
*ER stress/UPR*			
*ATF4*	Activating transcription factor 4	1.31	0.001
*ATF6*	Activating transcription factor 6	1.01	0.861
*BAK1*	BCL2 antagonist/killer 1	0.93	0.139
*BAX*	BCL2 associated X, apoptosis regulator	1.07	0.178
*CASP3*	Caspase 3	0.98	0.741
*CASP8*	Caspase 8	1.00	0.965
*CASP9*	Caspase 9	1.01	0.865
*CHAC1*	ChaC glutathione specific gamma-glutamylcyclotransferase 1	1.23	0.025
*DDIT3*	DNA damage inducible transcript 3	1.19	0.013
*DNAJC3*	DNAJ heat shock protein family (Hsp40) member C3	1.05	0.581
*EDEM1*	ER degradation enhancing alpha-mannosidase like protein 1	1.02	0.675
*HERPUD1*	Homocysteine inducible ER protein with ubiquitin like domain 1	1.32	0.044
*HSP90B1*	Heat shock protein 90 beta family member 1	1.14	0.372
*HSPA5*	Heat shock protein family A (Hsp70) member 5	1.53	0.050
*PDIA4*	Protein disulfide isomerase family A member 4	1.18	0.294
*PPP1R15A*	Protein phosphatase 1 regulatory subunit 15A	0.95	0.459
*TRIB3*	Tribbles pseudokinase 3	1.24	0.034
*XBP1*	X-box binding protein 1	1.21	0.112
Nrf2-dependent cytoprotection			
*CAT*	Catalase	−1.07	0.019
*GCLC*	Glutamate-cysteine ligase catalytic subunit	0.95	0.677
*GCLM*	Glutamate-cysteine ligase modifier subunit	0.92	0.281
*GPX1*	Glutathione peroxidase 1	0.98	0.858
*GPX3*	Glutathione peroxidase 3	2.90	0.046
*GSTA2*	Glutathione S-transferase alpha 2	−1.36	0.040
*HMOX1*	Heme oxygenase 1	1.09	0.325
*MT1A*	Metallothionein 1A	2.64	0.016
*MT2A*	Metallothionein 2A	1.89	0.045
*MT1E*	Metallothionein 1E	3.58	0.022
*MT3*	Metallothionein 3	0.98	0.608
*MT4*	Metallothionein 4	−1.11	0.031
*NFE2L2*	Nuclear factor, erythroid 2 like 2	1.04	0.420
*NQO1*	NAD(P)H quinone dehydrogenase 1	0.92	0.354
*SOD1*	Superoxide dismutase 1	0.98	0.634
*SRXN1*	Sulfiredoxin 1	1.08	0.295
*TXN*	Thioredoxin	0.92	0.221
*TXNRD1*	Thioredoxin reductase 1	1.01	0.866
*UGT1A1*	UDP glucuronosyltransferase family 1 member A1	0.91	0.061
*UGT1A6*	UDP glucuronosyltransferase family 1 member A6	1.24	0.452

*n* = 8 for each group.

**Table 5:** Plasma concentrations of parameters indicative of antioxidant status in cows with high and low hepatic *FGF21* expression at week 1 postpartum.

	Low *FGF21*	High *FGF21*	*p*-Value
Plasma			
α-tocopherol, µmol/L	6.12 ± 1.42	5.69 ± 1.96	0.619
γ-tocopherol, µmol/L	0.126 ± 0.057	0.102 ± 0.041	0.357
β-carotene, µmol/L	12.8 ± 3.83	12.8 ± 4.22	0.992
GSH, µmol/L	3.51 ± 4.23	4.23 ± 0.72	0.075
TEAC, mmol/L	3.54 ± 0.53	3.72 ± 0.41	0.458
TBARS, µmol/L	0.814 ± 0.159	0.781 ± 0.144	0.670
Protein carbonyls, µmol/L	0.326 ± 0.070	0.265 ± 0.053	0.093

GSH = glutathione; TEAC = trolox equivalent antioxidant capacity; TBARS = thiobarbituric acid-reactive substances; data are means ± SD, *n*  =  8 for each group.

**Table 6:** Concentrations of positive and negative acute phase proteins and oxylipids in plasma of cows with high and low hepatic *FGF21* expression at week 1 postpartum.

	Low *FGF21*	High *FGF21*	*p*-Value
Positive acute phase proteins			
HP, µg/mL	1489 ± 1723	367 ± 411	0.338
SAA, ng/mL	294 ± 63	372 ± 68	0.038
Negative acute phase proteins			
Albumin, g/dL	2.96 ± 0.37	2.90 ± 0.36	0.836
RBP4, ng/mL	1465 ± 439	1519 ± 463	0.820
Oxylipids, nmol/L			
9-HODE	22.5 ± 7.15	23.5 ± 3.42	0.727
13-HODE	21.4 ± 5.18	20.1 ± 3.04	0.568
12-HETE	6.56 ± 2.62	6.05 ± 1.59	0.875
15-HETE	1.53 ± 0.55	1.49 ± 0.34	0.864
LTB4	1.74 ± 0.67	2.02 ± 0.97	0.504
PGF2α	0.313 ± 0.201	0.350 ± 0.392	0.869

HETE = hydroxyeicosatetraenoic acid; HODE = hydroxy-octadecadienoic acid; HP = haptoglobin =; LTB4 = leukotriene B4; PGF2α = prostaglandin F2α; RBP4 = retinol-binding protein 4; SAA = serum amyloid A; data represent means ± SD, *n* = 8 for each group.

## Data Availability

The microarray data have been deposited in MIAME compliant format in the NCBI’s Gene Expression Omnibus public repository; GEO accession no. will be provided during review.

## Supplementary Materials

The following supporting information can be downloaded at: https://www.mdpi.com/article/10.3390/ani13010131/s1, Table S1: Characteristics of gene-specific primers; Table S2: Fold change (FC) and *p*-values of all differentially expressed transcripts, Table S3: Validation of gene chip data for selected differentially expressed transcripts by qPCR.
