# Supplementary material for: Performance and Metabolic, Inflammatory, and Oxidative Stress-Related Parameters in Early Lactating Dairy Cows with High and Low Hepatic FGF21 Expression
Source: Animals (Basel). 2022 Dec 29;13(1):131. doi: 10.3390/ani13010131 (PMC9817787; doi:10.3390/ani13010131)
Supplement: Supplementary file 1 [file animals-13-00131-s001.zip › animals-2052457-supplementary.pdf]

Table S1. Validation of gene chip data for selected differentially expressed transcripts by qPCR.

Gene symbol	mRNA description	Mean fold change[1]		*P*-value	
		Gene chip	qPCR	Gene chip	qPCR
FGF21	fibroblast growth factor 21	4.23	16.0	0.00001	0.0027
MT1E	metallothionein 1E	3.58	2.44	0.02151	0.0347
GPX3	glutathione peroxidase 3 (plasma)	2.90	1.75	0.04557	0.2142
MT1A	metallothionein-1A	2.64	2.84	0.01646	0.0447
STEAP4	STEAP family member 4	1.94	1.20	0.04495	0.3022
MT2A	metallothionein 2A	1.89	1.44	0.04520	0.1343
STK39	serine threonine kinase 39	1.81	1.54	0.00307	0.0626
PPP1R3C	protein phosphatase 1, regulatory subunit 3C	1.74	1.98	0.00644	0.0195
INHBE	inhibin, beta E	1.72	1.64	0.00014	0.0708
PKD1L3	polycystic kidney disease 1-like 3	1.67	1.32	0.01529	0.1226
BCL6	B-cell CLL/lymphoma 6	1.67	1.11	0.00271	0.3154
LBP	lipopolysaccharide binding protein	1.67	1.50	0.02337	0.2136
HAL	histidine ammonia-lyase	-1.91	-3.96	0.00167	0.0125
MFSD2A	major facilitator superfamily domain containing 2A	-1.89	-2.02	0.00185	0.0072

LPIN1	lipin 1	-1.77	-3.86	0.01014	0.0140
GSTM2	glutathione S-transferase M2	-1.75	-2.63	0.00998	0.0031
GSTM1	glutathione S-transferase M1	-1.68	-1.79	0.00878	0.0474
ASCL1	achaete-scute family bHLH transcription factor 1	-1.64	-2.58	0.02755	0.0199
ERRFI1	ERBB receptor feedback inhibitor 1	-1.60	-2.85	0.00724	0.0034
VWA3B	von Willebrand factor A domain containing 3B	-1.59	-2.50	0.01272	0.0221
ATP10B	ATPase, class V, type 10B	-1.57	-4.72	0.00278	0.0050
LGR5	leucine-rich repeat containing G protein-coupled receptor 5	-1.56	-1.69	0.01635	0.0369
ZPR1	ZPR1 zinc finger	-1.55	-2.93	0.00124	0.0001
ZNF37A	zinc finger protein 37A	-1.54	-1.64	0.00651	0.0737
AMDHD1	amidohydrolase domain containing 1	-1.53	-2.07	0.00206	0.0553

[1] Fold change was calculated from the signal log ratios as follows: $2^{\text{Signal log ratio}}$ if signal log ratio ≥ 0 and $(-1) \times 2^{-(\text{Signal log ratio})}$ if signal log ratio < 0. Signal log ratios were calculated from n = 8 microarrays per group.

Table S2 Characteristics of gene-specific primers.

Gene	Forward primer (from 5` to 3`)<br>Reverse primer (from 5` to 3`)	PCR product size (bp)	NCBI GenBank Accession no.	Annealing temperature
Reference genes				
EEF1A1	GTCAAAGATGTCCGTVCGTGGC<br>TGGCAGCGTCACCAGATTTCA	253	NM_174535.2	60
H3F3A	CTGTGGCACTCCGTGAAATTA<br>ACTTGCCTCCTGCAAA.GCAC	154	NM_001014389.2	60
RPL12	GGCAACTGGTGATTGGAAGG<br>TCTGCTTCTTTCTGTCCCTTGG	143	NM_205797.1	60
Target genes				
FGF21	TGTGGGTCCCTGTGCTGGCT<br>CCACTGTGCCATCGGCCCTG	171	XM_001789587.1	65
MT1E	TTGCTTTGGACGACCACACTTC<br>ATTGATACCTGGGGCAGGCTC	246	NM_001114857.2	63
GPX3	ACC ACC GCA CCA CGG TCA AC<br>GCC CGT GTG GTG GAC TTG GG	127	NM_174077.3	63
MT1A	ATCCGACCAGTGGATCTGCTTTGCC<br>AGACACAGCCCTGGGCACACT	209	NM_001040492.2	63
STEAP4	AGCACCTCTCCCGCAGTTATG<br>GGACATCCCAGGGGTTCGAC	183	XM_002686859.5	60
MT2A	ACCCTCGCCATCCTTTGCTC<br>AGGGATGTAACAAACGGGTCAGG	339	NM_001075140.1	60
STK39	AGGAGGTTATCGGCAGTGGC<br>TGGGATGGCTGCACTGACTC	162	NM_001075826.1	60
PPP1R3C	TGCCATTGACCTACCCTCCG	142	NM_001076164.2	60

	CCCGTCAGGCTTCCACTGTA			
INHBE	TTGCTGTCCTCACAGACTCCT	101	NM_001205842.1	60
	CCAGAGGCGAGCATGATACA			
PKD1L3	AGTGGCTAGACCATTGCACC	254	XM_010814701	60
	CTGCTGTTTCAACTGGCGAC			
BCL6	TCATTTTAGAGTGCTCATTTGGTTT	248	NM_001206450.1	60
	TGCTGTAGAACAGGCCACTG			
LBP	TGCGCAAGAGGATACTGAGAC	218	NM_001038674.2	60
	TCGGAACCTGGACTCGATCT			
HAL	CTAAGCCCTGAGAGATGCCG	268	NM_001105440.1	60
	CATGAGCTGCGAGGACGTAT			
MFSD2A	CGGTCCAGGTGAAGAAGGAA	231	NM_001101959.1	60
	TAATGCAGAAGCCCACCAGG			
LPIN1	CTTCAGTTTCCCGACCGTCA	142	NM_001206156.2	60
	TTGTGTGGCGAGGACTTTGT			
GSTM2	CTCCCCCACCCCACATTATC	242	XM_005204221.4	60
	CCAGGAGGGAATAAGCAGGTG			
GSTM1	GAGAAGACAAGTTTCAAGCCCA	193	NM_175825.3	60
	CTGTCATAGTCGGGAGCATCT			
ASCL1	CCCCCAACTACTCCAACGAC	181	XM_002687640.5	60
	CCCTGCTTCCGAAGTCCATT			
ERRFI1	GCGAGTTTGAGAACGGCTTT	292	NM_001077930.1	60
	CTCTTGACAGGGGTTAGCGG			
VWA3B	GAAGCTGGCAGCATATTGGC	112	XM_015473358.2	60
	CCCAGGCGTCTTCTGGATTT			
ATP10B	GGGGTGGAGGAATTGTGTTCA	190	XM_010798068	60
	CTTGGGCCCTTCTTGGTTTC			
LGR5	TGCAGTCAGGACACTCTCCA	192	NM_001192520.3	60

	GCGCCATTCAAAGTCAGTGT			
ZPR1	GCCACCACAAGGATTCCAGA	144	NM_001038504.1	60
	AGCTTCTTCGTTCGCCCTTC			
ZNF37A	AGGAGAGGATTCCGGAGCTT	153	XM_010801271	60
	AAACGGAGTCAGAGCCACAC			
AMDHD1	CGGCGAGCGCTTCCTAAC	261	NM_001098872.2	60
	GTGGCTCCTGCCAACTTCAT			

Table S3 Fold change (FC) and *P*-value of all differentially expressed transcripts*

Gene Symbol	Gene Description	FGF21 high vs. FGF21 low	
		FC	*P*-value
FGF21	fibroblast growth factor 21	4.23	0.00001
MT1E	metallothionein 1E	3.58	0.02151
GPX3	glutathione peroxidase 3 (plasma)	2.90	0.04557
MT1A	metallothionein-1A	2.64	0.01646
MT1E	metallothionein 1E	2.11	0.04023
MIR708	microRNA mir-708	2.00	0.01304
STEAP4	STEAP family member 4	1.94	0.04495
MT2A	metallothionein 2A	1.89	0.04520
SLC22A7	solute carrier family 22 (organic anion transporter), member 7	1.85	0.00004
STK39	serine threonine kinase 39	1.81	0.00307
PPP1R3C	protein phosphatase 1, regulatory subunit 3C	1.74	0.00644
INHBE	inhibin, beta E	1.72	0.00014
SPIDR	scaffolding protein involved in DNA repair	1.70	0.02979
PKD1L3	polycystic kidney disease 1-like 3	1.67	0.01529
BCL6	B-cell CLL/lymphoma 6	1.67	0.00271
LBP	lipopolysaccharide binding protein	1.67	0.02337
LOC788334	apolipoprotein L3	1.66	0.02124
SAA4	serum amyloid A4, constitutive	1.64	0.04790
WWC1	WW and C2 domain containing 1	1.64	0.00131
MANF	mesencephalic astrocyte-derived neurotrophic factor	1.62	0.04617
PFKFB3	6-phosphofructo-2-kinase/fructose-2,6-biphosphatase 3	1.62	0.00081
SLC25A33	solute carrier family 25 (pyrimidine nucleotide carrier), member 33	1.60	0.00269
PPP1R3B	protein phosphatase 1, regulatory subunit 3B	1.59	0.01829
INHBA	inhibin, beta A	1.57	0.00081
PFKFB2	6-phosphofructo-2-kinase/fructose-2,6-biphosphatase 2	1.54	0.00229
DCDC2	doublecortin domain containing 2	1.54	0.01498
HSPA5	heat shock 70kDa protein 5 (glucose-regulated protein, 78kDa)	1.53	0.04974
ARRDC3	arrestin domain containing 3	1.49	0.00782
MID1IP1	MID1 interacting protein 1	1.49	0.00076
LOC101908417	complement C4-like	1.47	0.02331
SERPINA3-8	SERPINA3-8	1.47	0.03278
SGPL1	sphingosine-1-phosphate lyase 1	1.47	0.00895
LOC104974345	glycine N-acyltransferase-like	1.47	0.02243
CPQ	carboxypeptidase Q	1.46	0.02643
DNAJB11	DnaJ (Hsp40) homolog, subfamily B, member 11	1.44	0.01839
MIR2284R	microRNA mir-2284r	1.42	0.01088

S100A12	S100 calcium binding protein A12	1.42	0.00605
PSPH	phosphoserine phosphatase	1.42	0.00009
LOC785811	olfactory receptor 12D2	1.41	0.01320
LOC100336476	granulocyte-macrophage colony-stimulating factor receptor subunit alpha	1.41	0.01720
TIMD4	T-cell immunoglobulin and mucin domain containing 4	1.40	0.03793
CRELD2	cysteine-rich with EGF-like domains 2	1.40	0.01898
LOC104968675	apolipoprotein L3-like	1.39	0.02347
G0S2	G0/G1 switch 2	1.39	0.00901
SLC39A14	solute carrier family 39 (zinc transporter), member 14	1.39	0.00064
LOC786942	uncharacterized LOC786942	1.39	0.03517
LOC100300896	sulfotransferase 1C4	1.39	0.01453
LEAP2	liver expressed antimicrobial peptide 2	1.38	0.00339
SLC20A1	solute carrier family 20 (phosphate transporter), member 1	1.37	0.00127
SLC1A1	solute carrier family 1 (neuronal/epithelial high affinity glutamate transporter, system Xag), member 1	1.37	0.01857
ATP6V0D2	ATPase, H+ transporting, lysosomal 38kDa, V0 subunit d2	1.37	0.01956
ASNS	asparagine synthetase (glutamine-hydrolyzing)	1.37	0.03515
MIR874	microRNA mir-874	1.36	0.01771
MAMSTR	MEF2 activating motif and SAP domain containing transcriptional regulator	1.36	0.00082
CITED4	Cbp/p300-interacting transactivator, with Glu/Asp-rich carboxy-terminal domain, 4	1.36	0.00001
ZCCHC10	zinc finger, CCHC domain containing 10	1.36	0.03243
ALDH1L2	aldehyde dehydrogenase 1 family, member L2	1.35	0.02364
LARP1B	La ribonucleoprotein domain family, member 1B	1.35	0.00307
GZMK	granzyme K (granzyme 3; tryptase II)	1.35	0.02657
GRAMD4	GRAM domain containing 4	1.34	0.00752
C15H11orf96	chromosome 15 open reading frame, human C11orf96	1.34	0.00691
SLC16A1	solute carrier family 16 (monocarboxylate transporter), member 1	1.34	0.00055
MEIOB	meiosis specific with OB domains	1.34	0.00973
CS	citrate synthase	1.34	0.00356
GLUL	glutamate-ammonia ligase	1.33	0.03033
HDHD3	haloacid dehalogenase-like hydrolase domain containing 3	1.33	0.00211
MICAL2	microtubule associated monooxygenase, calponin and LIM domain containing 2	1.33	0.03967
SELK	selenoprotein K	1.32	0.00698
UAP1	UDP-N-acetylglucosamine pyrophosphorylase 1	1.32	0.00463
PPL	periplakin	1.32	0.00366

HERPUD1	homocysteine-inducible, endoplasmic reticulum stress-inducible, ubiquitin-like domain member 1	1.32	0.04380
EPHA2	EPH receptor A2	1.32	0.00227
MIR2372	microRNA mir-2372	1.32	0.03830
MDM4	MDM4, p53 regulator	1.32	0.00673
PLK2	polo-like kinase 2	1.32	0.01037
LOC785803	lysozyme 14D	1.32	0.02452
RAP1GAP	RAP1 GTPase activating protein	1.31	0.00384
HES1	hes family bHLH transcription factor 1	1.31	0.01160
NPR3	natriuretic peptide receptor 3	1.31	0.02876
XPO1	exportin 1	1.31	0.01228
ATF4	activating transcription factor 4	1.31	0.00130
RORC	RAR-related orphan receptor C	1.31	0.02388
IL4R	interleukin 4 receptor	1.31	0.00097
TMEM86A	transmembrane protein 86A	1.31	0.02259
WDR76	WD repeat domain 76	1.30	0.02358
LOC782221	olfactory receptor 8B3	1.30	0.00747
AGPAT9	1-acylglycerol-3-phosphate O-acyltransferase 9	1.30	0.02074
LYVE1	lymphatic vessel endothelial hyaluronan receptor 1	1.30	0.01631
KIF23	kinesin family member 23	1.30	0.04910
LOC101904076	glutathione S-transferase theta-1-like	1.30	0.02855
LOC784376	olfactory receptor 52D1	1.29	0.04756
LOC528329	uncharacterized LOC528329	1.29	0.01205
LOC787740	olfactory receptor 2A12	1.29	0.01747
ENTPD7	ectonucleoside triphosphate diphosphohydrolase 7	1.29	0.01661
NFIL3	nuclear factor, interleukin 3 regulated	1.29	0.01156
ALPI	alkaline phosphatase, intestinal	1.29	0.03577
C6H4orf19	chromosome 6 open reading frame, human C4orf19	1.29	0.01004
STK38	serine/threonine kinase 38	1.28	0.00037
HS3ST3A1	heparan sulfate (glucosamine) 3-O-sulfotransferase 3A1	1.28	0.02186
LOC619125	putative SEC14-like protein 6	1.28	0.02206
ZFAND2A	zinc finger, AN1-type domain 2A	1.28	0.01425
RGL1	ral guanine nucleotide dissociation stimulator-like 1	1.28	0.00155
CISD2	CDGSH iron sulfur domain 2	1.28	0.01803
STARD6	StAR-related lipid transfer (START) domain containing 6	1.28	0.02658
ASPHD2	aspartate beta-hydroxylase domain containing 2	1.28	0.02626
DENND2A	DENN/MADD domain containing 2A	1.28	0.04855
LOC101907658	60S ribosomal protein L22-like 1	1.27	0.02534
SOWAHB	sosondowah ankyrin repeat domain family member B	1.27	0.00575
LOC784623	olfactory receptor 5B12	1.27	0.01102
BOSTAUV1R403	vomeronasal 1 receptor bosTauV1R403	1.27	0.00838
SH3BP2	SH3-domain binding protein 2	1.27	0.02830
UGDH	UDP-glucose 6-dehydrogenase	1.27	0.00037

ZNF608	zinc finger protein 608	1.27	0.02961
KRT6B	keratin 6B	1.27	0.01084
CCDC79	coiled-coil domain containing 79	1.26	0.02048
CDKN1A	cyclin-dependent kinase inhibitor 1A (p21, Cip1)	1.26	0.04303
USP50	ubiquitin specific peptidase 50	1.26	0.00089
LOC786352	apolipoprotein L3	1.26	0.01865
MAP1LC3A	microtubule-associated protein 1 light chain 3 alpha	1.26	0.00757
CCDC134	coiled-coil domain containing 134	1.26	0.03557
WFS1	Wolfram syndrome 1 (wolframin)	1.26	0.03334
HPX	hemopexin	1.26	0.02358
RIOK1	RIO kinase 1	1.26	0.00319
RNF144B	ring finger protein 144B	1.26	0.00606
TBC1D9	TBC1 domain family, member 9 (with GRAM domain)	1.25	0.03029
C8H9orf89	chromosome 8 open reading frame, human C9orf89	1.25	0.00062
RRH	retinal pigment epithelium-derived rhodopsin homolog	1.25	0.00829
HAMP	hepcidin antimicrobial peptide	1.25	0.04570
FSTL1	follistatin-like 1	1.25	0.02243
ATP8B1	ATPase, aminophospholipid transporter, class I, type 8B, member 1	1.25	0.01479
TMEM41B	transmembrane protein 41B	1.25	0.01878
RNF43	ring finger protein 43	1.25	0.00907
CDC42EP3	CDC42 effector protein (Rho GTPase binding) 3	1.25	0.02708
FXYD6	FXYD domain containing ion transport regulator 6	1.25	0.00515
FBXO21	F-box protein 21	1.25	0.01659
ZHX3	zinc fingers and homeoboxes 3	1.24	0.00127
HSPB8	heat shock 22kDa protein 8	1.24	0.00307
TRIB3	tribbles pseudokinase 3	1.24	0.03366
IRF1	interferon regulatory factor 1	1.24	0.04070
MIR1197	microRNA mir-1197	1.24	0.04820
RNF144A	ring finger protein 144A	1.24	0.00219
CDC37L1	cell division cycle 37-like 1	1.24	0.01153
C3H1orf52	chromosome 3 open reading frame, human C1orf52	1.24	0.02196
FYN	FYN proto-oncogene, Src family tyrosine kinase	1.24	0.00232
CLDN15	claudin 15	1.23	0.03785
FNDC3B	fibronectin type III domain containing 3B	1.23	0.03420
CHAC1	ChaC glutathione-specific gamma-glutamylcyclotransferase 1	1.23	0.02543
IRF2BPL	interferon regulatory factor 2 binding protein-like	1.23	0.00737
ZMYND10	zinc finger, MYND-type containing 10	1.23	0.00808
FGL1	fibrinogen-like 1	1.23	0.01453
CTPS1	CTP synthase 1	1.23	0.02064
SLC4A7	solute carrier family 4, sodium bicarbonate cotransporter, member 7	1.23	0.00159
LITAF	lipopolysaccharide-induced TNF factor	1.23	0.02406

GALNTL6	polypeptide N-acetylgalactosaminyltransferase-like 6	1.23	0.00361
OR1L6	olfactory receptor, family 1, subfamily L, member 6	1.23	0.01562
SH2D1A	SH2 domain containing 1A	1.23	0.00434
ID1	inhibitor of DNA binding 1, dominant negative helix-loop-helix protein	1.23	0.00571
GZMB	granzyme B (granzyme 2, cytotoxic T-lymphocyte-associated serine esterase 1)	1.22	0.02274
TTC7A	tetratricopeptide repeat domain 7A	1.22	0.00660
UBE2L6	ubiquitin-conjugating enzyme E2L 6	1.22	0.02858
FAM134B	family with sequence similarity 134, member B	1.22	0.00625
LOC785981	olfactory receptor 7A17	1.22	0.01594
MTHFD1L	methylenetetrahydrofolate dehydrogenase (NADP+ dependent) 1-like	1.22	0.01228
CAGE1	cancer antigen 1	1.22	0.03954
LOC513333	olfactory receptor 151	1.22	0.02806
SLC38A7	solute carrier family 38, member 7	1.22	0.03120
CNOT1	CCR4-NOT transcription complex, subunit 1	1.22	0.02638
BCAR3	breast cancer anti-estrogen resistance 3	1.22	0.00482
SLC30A1	solute carrier family 30 (zinc transporter), member 1	1.22	0.00688
CEBPA	CCAAT/enhancer binding protein (C/EBP), alpha	1.22	0.01341
LRCH1	leucine-rich repeats and calponin homology (CH) domain containing 1	1.21	0.00884
SKIL	SKI-like proto-oncogene	1.21	0.01870
BIN2	bridging integrator 2	1.21	0.01017
MIR487A	microRNA mir-487a	1.21	0.04780
RCN2	reticulocalbin 2, EF-hand calcium binding domain	1.21	0.02028
SDC4	syndecan 4	1.21	0.01125
LRAT	lecithin retinol acyltransferase (phosphatidylcholine--retinol O-acyltransferase)	1.21	0.01511
MIR144	microRNA mir-144	1.21	0.02171
PSMG4	proteasome (prosome, macropain) assembly chaperone 4	1.21	0.02158
LOC515150	apolipoprotein R	1.21	0.03285
CORO1C	coronin, actin binding protein, 1C	1.21	0.00772
PARM1	prostate androgen-regulated mucin-like protein 1	1.21	0.00159
C6H4orf32	chromosome 6 open reading frame, human C4orf32	1.21	0.00220
IHH	indian hedgehog	1.21	0.02710
MIR208A	microRNA mir-208a	1.21	0.04147
GPRC5C	G protein-coupled receptor, class C, group 5, member C	1.21	0.00374
AGFG1	ArfGAP with FG repeats 1	1.21	0.01705
TMEM50B	transmembrane protein 50B	1.21	0.03759
PLAGL1	pleiomorphic adenoma gene-like 1	1.21	0.01149
FLOT2	flotillin 2	1.21	0.01589
LOC783070	60S ribosomal protein L5 pseudogene	-1.21	0.01177

PRR29	proline rich 29	-1.21	0.00032
BVD1.15	T cell receptor delta chain variable region BVd1.15	-1.21	0.01706
BCL11B	B-cell CLL/lymphoma 11B (zinc finger protein)	-1.21	0.03007
SMO	smoothened, frizzled class receptor	-1.21	0.00139
ATP8A1	ATPase, aminophospholipid transporter (APLT), class I, type 8A, member 1	-1.21	0.00893
FRMPD3	FERM and PDZ domain containing 3	-1.21	0.01367
NPAS4	neuronal PAS domain protein 4	-1.21	0.00828
TMEM38B	transmembrane protein 38B	-1.21	0.02574
GAS2	growth arrest-specific 2	-1.21	0.02180
TTC21B	tetratricopeptide repeat domain 21B	-1.21	0.04557
PPFIA3	protein tyrosine phosphatase, receptor type, f polypeptide (PTPRF), interacting protein (liprin), alpha 3	-1.21	0.00022
SLC51B	solute carrier family 51, beta subunit	-1.21	0.04918
HRH3	histamine receptor H3	-1.21	0.01832
KRT26	keratin 26	-1.21	0.04773
STARD13	StAR-related lipid transfer (START) domain containing 13	-1.21	0.00033
FAAH	fatty acid amide hydrolase	-1.21	0.01007
IFNAH	interferon alpha H	-1.21	0.02866
LOC100300010	olfactory receptor 8S1	-1.21	0.01270
LONRF1	LON peptidase N-terminal domain and ring finger 1	-1.21	0.01748
SEL1L3	sel-1 suppressor of lin-12-like 3 (C. elegans)	-1.21	0.04107
C5H12orf71	chromosome 5 open reading frame, human C12orf71	-1.21	0.02732
ANTXR2	anthrax toxin receptor 2	-1.21	0.01263
LOC101906359	Y-box-binding protein 3-like	-1.21	0.03913
MMP12	matrix metallopeptidase 12 (macrophage elastase)	-1.21	0.02363
G6PC	glucose-6-phosphatase, catalytic subunit	-1.21	0.01044
ASPRV1	aspartic peptidase, retroviral-like 1	-1.22	0.00223
GRIN2C	glutamate receptor, ionotropic, N-methyl D-aspartate 2C	-1.22	0.00711
PPM1K	protein phosphatase, Mg2+/Mn2+ dependent, 1K	-1.22	0.01647
PROX2	prospero homeobox 2	-1.22	0.00111
MIR184	microRNA mir-184	-1.22	0.04469
ENO2	enolase 2 (gamma, neuronal)	-1.22	0.02159
CLDN1	claudin 1	-1.22	0.02000
LOC100848786	lipid phosphate phosphatase-related protein type 5	-1.22	0.01549
SCN9A	sodium channel, voltage-gated, type IX, alpha subunit	-1.22	0.04638
HCRTR2	hypocretin (orexin) receptor 2	-1.22	0.04439
ZNF319	zinc finger protein 319	-1.22	0.00919
CCDC7	coiled-coil domain containing 7	-1.22	0.01962
DMP1	dentin matrix acidic phosphoprotein 1	-1.22	0.00336
INSIG2	insulin induced gene 2	-1.22	0.03109
GJA3	gap junction protein, alpha 3, 46kDa	-1.22	0.00165

SGCZ	sarcoglycan, zeta	-1.22	0.01501
KRT1	keratin 1	-1.22	0.00436
IP6K2	inositol hexakisphosphate kinase 2	-1.22	0.02957
ATL1	atlastin GTPase 1	-1.22	0.01418
LOC538872	dual specificity phosphatase 18-like	-1.22	0.02587
CRABP2	cellular retinoic acid binding protein 2	-1.22	0.00698
PBLD	phenazine biosynthesis-like protein domain containing	-1.23	0.02703
LOC787011	uncharacterized protein CXorf66 homolog	-1.23	0.00691
RIMBP3B	RIMS binding protein 3B	-1.23	0.02487
LOC100300695	intelectin-2	-1.23	0.01674
ZNF750	zinc finger protein 750	-1.23	0.01019
NT5DC1	5-nucleotidase domain containing 1	-1.23	0.01786
LOC504344	olfactory receptor 4F15	-1.23	0.02552
LOC784187	olfactory receptor 51L1	-1.23	0.04964
RAB3C	RAB3C, member RAS oncogene family	-1.23	0.00064
LOC101906200	ninjurin-1 pseudogene	-1.23	0.01542
LOC537188	UDP-glucuronosyltransferase 3A1-like	-1.24	0.00249
AGL	amylo-alpha-1, 6-glucosidase, 4-alpha-glucanotransferase	-1.24	0.01408
RBMS2	RNA binding motif, single stranded interacting protein 2	-1.24	0.01436
MBNL3	muscleblind-like splicing regulator 3	-1.24	0.02344
LOC784679	peptidyl-prolyl cis-trans isomerase A	-1.24	0.03099
FAM71B	family with sequence similarity 71, member B	-1.24	0.00498
LOC100125266	alkaline phosphatase-like	-1.24	0.04400
OR2G6	olfactory receptor, family 2, subfamily G, member 6	-1.24	0.00475
PDK2	pyruvate dehydrogenase kinase, isozyme 2	-1.24	0.01237
FST	follistatin	-1.24	0.00289
MYBL2	v-myb avian myeloblastosis viral oncogene homolog-like 2	-1.24	0.00132
LOC785899	olfactory receptor 1440	-1.24	0.02496
AQP9	aquaporin 9	-1.24	0.00390
GABRQ	gamma-aminobutyric acid (GABA) A receptor, theta	-1.24	0.00578
TMEM184C	transmembrane protein 184C	-1.25	0.04891
OPN1SW	opsin 1 (cone pigments), short-wave-sensitive	-1.25	0.01030
SLC16A10	solute carrier family 16 (aromatic amino acid transporter), member 10	-1.25	0.00681
MIR2475	microRNA mir-2475	-1.25	0.01011
MIR1603	microRNA mir-1603	-1.25	0.00738
LOC511642	olfactory receptor 18	-1.25	0.03583
LOC788998	olfactory receptor, family 9, subfamily K, member 2-like	-1.25	0.01252
PLCG2	phospholipase C, gamma 2 (phosphatidylinositol-specific)	-1.25	0.00039

ABCB11	ATP-binding cassette, sub-family B (MDR/TAP), member 11	-1.25	0.01637
ELL2	elongation factor, RNA polymerase II, 2	-1.25	0.02513
OTC	ornithine carbamoyltransferase	-1.25	0.00401
TMEM144	transmembrane protein 144	-1.25	0.04843
FIGF	c-fos induced growth factor (vascular endothelial growth factor D)	-1.25	0.00748
TAT	tyrosine aminotransferase	-1.25	0.03960
LOC100848572	keratin-associated protein 4-7-like	-1.25	0.03490
MIR23A	microRNA mir-23a	-1.26	0.01646
SERPINE3	serpin peptidase inhibitor, clade E (nexin, plasminogen activator inhibitor type 1), member 3	-1.26	0.00217
MIR105B	microRNA mir-105b	-1.26	0.02745
HSD11B1	hydroxysteroid (11-beta) dehydrogenase 1	-1.26	0.00165
LOC101903205	alpha-2-macroglobulin receptor-associated protein pseudogene	-1.26	0.01288
SALL1	spalt-like transcription factor 1	-1.26	0.03715
OR51E1	olfactory receptor, family 51, subfamily E, member 1	-1.26	0.00028
LOC785144	olfactory receptor, family 51, subfamily E, member 2-like	-1.26	0.04072
JAKMIP2	janus kinase and microtubule interacting protein 2	-1.26	0.00166
IQCF1	IQ motif containing F1	-1.26	0.01104
LOC100297248	Ig kappa chain V-III region PC 7210	-1.26	0.02055
LOC504570	olfactory receptor 1L4	-1.27	0.03432
MYC	v-myc avian myelocytomatosis viral oncogene homolog	-1.27	0.04674
C18H16orf87	chromosome 18 open reading frame, human C16orf87	-1.27	0.00823
LOC104975671	ubiquitin D-like	-1.27	0.00977
GLP1R	glucagon-like peptide 1 receptor	-1.27	0.01386
MAT1A	methionine adenosyltransferase I, alpha	-1.27	0.00005
ZNF883	zinc finger protein 883	-1.27	0.01005
RASSF6	Ras association (RalGDS/AF-6) domain family member 6	-1.27	0.03790
KCNJ2	potassium inwardly-rectifying channel, subfamily J, member 2	-1.27	0.04198
LOC101905711	heme-binding protein 1	-1.28	0.03768
LOC787624	ADAMTS-like protein 3	-1.28	0.01883
LOC783144	60S ribosomal protein L13 pseudogene	-1.28	0.00231
LOC787129	glycine cleavage system H protein, mitochondrial	-1.28	0.00175
ARHGEF9	Cdc42 guanine nucleotide exchange factor (GEF) 9	-1.28	0.03088
GRIK3	glutamate receptor, ionotropic, kainate 3	-1.28	0.00036
LOC504233	olfactory receptor 2A1/2A42	-1.28	0.04420
LOC789943	olfactory receptor 13C2	-1.28	0.02491
LOC100299372	olfactory receptor 2S2	-1.28	0.02331
STXBP6	syntaxin binding protein 6 (amisyn)	-1.28	0.02821

LOC783917	olfactory receptor 187	-1.28	0.02245
LOC785712	olfactory receptor 12D2	-1.29	0.02805
GSAP	gamma-secretase activating protein	-1.29	0.04778
LOC751804	WC1 isolate DV100	-1.29	0.01612
SOX5	SRY (sex determining region Y)-box 5	-1.29	0.00356
BPIFA2B	BPI fold containing family A, member 2B	-1.29	0.00986
PIGP	phosphatidylinositol glycan anchor biosynthesis, class P	-1.29	0.04820
SNCAIP	synuclein, alpha interacting protein	-1.29	0.01033
MIR27B	microRNA mir-27b	-1.29	0.00498
LOC522775	olfactory receptor 8J2	-1.29	0.00738
LOC618034	olfactory receptor 12D3	-1.29	0.02320
UHRF1	ubiquitin-like with PHD and ring finger domains 1	-1.29	0.01016
FGF12	fibroblast growth factor 12	-1.29	0.00191
SLC25A30	solute carrier family 25, member 30	-1.29	0.01297
LOC100337180	eukaryotic translation elongation factor 1 alpha 1 pseudogene	-1.30	0.03742
IVD	isovaleryl-CoA dehydrogenase	-1.30	0.00096
LOC788079	olfactory receptor 2L8	-1.30	0.04603
FOXP1	forkhead box P1	-1.30	0.02469
NECAB2	N-terminal EF-hand calcium binding protein 2	-1.30	0.02836
LOC100337053	multidrug resistance-associated protein 4-like	-1.30	0.04205
LOC617135	interferon omega-1	-1.31	0.00591
TDH	L-threonine dehydrogenase	-1.31	0.00025
MIR940	microRNA mir-940	-1.31	0.00153
PNP	purine nucleoside phosphorylase	-1.31	0.00171
EDNRA	endothelin receptor type A	-1.31	0.01341
ADH4	alcohol dehydrogenase 4 (class II), pi polypeptide	-1.31	0.00334
OR5D18	olfactory receptor, family 5, subfamily D, member 18	-1.31	0.02317
ZNF679	zinc finger protein 679	-1.32	0.00631
FBP1	fructose-1,6-bisphosphatase 1	-1.32	0.00134
SMARCA1	SWI/SNF related, matrix associated, actin dependent regulator of chromatin, subfamily a, member 1	-1.32	0.00123
LOC515551	protein DDI1 homolog 2	-1.32	0.00613
BOSTAUV1R414	vomeronasal 1 receptor bosTauV1R414	-1.32	0.02124
LOC515540	olfactory receptor 1E2	-1.32	0.04412
CITED1	Cbp/p300-interacting transactivator, with Glu/Asp-rich carboxy-terminal domain, 1	-1.32	0.00302
LOC508595	olfactory receptor 51G2	-1.33	0.03560
PCTP	phosphatidylcholine transfer protein	-1.33	0.00697
LOC531650	H2B histone family, member W, testis-specific-like pseudogene	-1.33	0.01198
LOC782554	putative olfactory receptor 2W6	-1.33	0.04590
LOC100298264	uncharacterized LOC100298264	-1.33	0.01968
BTG2	BTG family, member 2	-1.33	0.02334
LOC513329	major allergen Equ c 1	-1.34	0.01500

GNAT3	guanine nucleotide binding protein, alpha transducing 3	-1.34	0.00017
TDRD10	tudor domain containing 10	-1.34	0.04056
GYS2	glycogen synthase 2 (liver)	-1.34	0.01646
LOC524160	olfactory receptor 2T3	-1.34	0.01028
LOC523582	olfactory receptor 12	-1.34	0.03900
LOC100298605	olfactory receptor 5T2	-1.34	0.03626
GLYATL3	glycine-N-acyltransferase-like 3	-1.34	0.00768
ARG2	arginase 2	-1.35	0.00066
MIR103-2	microRNA mir-103-2	-1.35	0.04396
GSTA2	glutathione S-transferase alpha 2	-1.36	0.03955
LOC100336901	olfactory receptor 8J3	-1.36	0.04626
SLC25A15	solute carrier family 25 (mitochondrial carrier; ornithine transporter) member 15	-1.36	0.00889
MIR339B	microRNA mir-339b	-1.37	0.02691
LOC510712	olfactory receptor 7A17	-1.37	0.00005
BICC1	BicC family RNA binding protein 1	-1.38	0.00169
LOC523530	nuclear RNA export factor 3	-1.38	0.01477
ADRA1A	adrenoceptor alpha 1A	-1.38	0.00143
MOGAT1	monoacylglycerol O-acyltransferase 1	-1.38	0.00184
LOC788220	olfactory receptor 6C76	-1.38	0.00570
ADRB2	adrenoceptor beta 2, surface	-1.39	0.00079
PCDH19	protocadherin 19	-1.39	0.00243
SLC4A4	solute carrier family 4 (sodium bicarbonate cotransporter), member 4	-1.39	0.03198
DBP	D site of albumin promoter (albumin D-box) binding protein	-1.40	0.01658
CCRN4L	CCR4 carbon catabolite repression 4-like (S. cerevisiae)	-1.40	0.01715
ATP10B	ATPase, class V, type 10B	-1.40	0.01451
ACMSD	aminocarboxymuconate semialdehyde decarboxylase	-1.40	0.04530
LOC786363	tumor susceptibility gene 101 protein pseudogene	-1.41	0.00950
LOC100138638	retinol dehydrogenase 16-like	-1.41	0.01970
IRS2	insulin receptor substrate 2	-1.41	0.00418
LOC104976490	complement factor H-like	-1.41	0.00587
LRP2	low density lipoprotein receptor-related protein 2	-1.42	0.00660
VNN1	vanin 1	-1.42	0.04659
LOC100337097	cationic amino acid transporter 3-like	-1.42	0.01886
ARG1	arginase 1	-1.42	0.01348
PPARGC1A	peroxisome proliferator-activated receptor gamma, coactivator 1 alpha	-1.42	0.01809
LOC618124	olfactory receptor, family 1, subfamily E, member 2-like	-1.44	0.04559
LOC527057	multidrug resistance-associated protein 4-like	-1.45	0.03897
LOC531237	olfactory receptor 4C13	-1.45	0.00140

SLC7A2	solute carrier family 7 (cationic amino acid transporter, y+ system), member 2	-1.46	0.00679
SOCS2	suppressor of cytokine signaling 2	-1.47	0.02636
DIRAS3	DIRAS family, GTP-binding RAS-like 3	-1.47	0.00187
TDO2	tryptophan 2,3-dioxygenase	-1.47	0.00534
TLE1	transducin-like enhancer of split 1 (E(sp1) homolog, Drosophila)	-1.48	0.00092
XPNPEP2	X-prolyl aminopeptidase (aminopeptidase P) 2, membrane-bound	-1.49	0.03487
PPAP2B	phosphatidic acid phosphatase type 2B	-1.49	0.00067
LURAP1L	leucine rich adaptor protein 1-like	-1.49	0.01546
TEX12	testis expressed 12	-1.51	0.02480
RALGPS2	Ral GEF with PH domain and SH3 binding motif 2	-1.53	0.02406
AMDHD1	amidohydrolase domain containing 1	-1.53	0.00206
ZNF37A	zinc finger protein 37A	-1.54	0.00651
ZPR1	ZPR1 zinc finger	-1.55	0.00124
LGR5	leucine-rich repeat containing G protein-coupled receptor 5	-1.56	0.01635
ATP10B	ATPase, class V, type 10B	-1.57	0.00278
VWA3B	von Willebrand factor A domain containing 3B	-1.58	0.01272
GLS2	glutaminase 2 (liver, mitochondrial)	-1.59	0.00007
ERRFI1	ERBB receptor feedback inhibitor 1	-1.60	0.00724
ASCL1	achaete-scute family bHLH transcription factor 1	-1.64	0.02755
GSTM1	glutathione S-transferase M1	-1.68	0.00878
GSTM2	glutathione S-transferase M2	-1.75	0.00998
LPIN1	lipin 1	-1.77	0.01014
MFSD2A	major facilitator superfamily domain containing 2A	-1.89	0.00185
HAL	histidine ammonia-lyase	-1.91	0.00167
PDK4	pyruvate dehydrogenase kinase, isozyme 4	-1.93	0.00002

*Filter criteria: FC > 1.2 and < -1.2 and P < 0.05 between FGF21 high vs. FGF21 low group. FCs were calculated from the signal log ratios, which were calculated from n = 8 microarrays per group.
